# Supplementary material for: Process evaluation protocol for the I-WOTCH study: an opioid tapering support programme for people with chronic non-malignant pain
Source: BMJ Open. 2019 Oct 10;9(10):e028998. doi: 10.1136/bmjopen-2019-028998 (PMC6797361; doi:10.1136/bmjopen-2019-028998)
Supplement: Supplementary data [file bmjopen-2019-028998supp001.pdf]

Group venue: ______________________________

Date: ______________________________

I-WOTCH Feedback form

Thank you for attending the I-WOTCH course. Please complete the feedback and short questionnaire below. This information will be used to evaluate the support programme. Please note that these forms are anonymous and will be sent back to the study team.

1. Were the aims of the course made clear? Please circle one

Yes *No* *Don't know*

2. What were the three most useful things on this course?

1)..

2)..

3)..

3. What three things would you suggest to make this course better for future participants?

1) ....

2) ....

3) ....

4. How confident do you feel that the course content will help you personally? *(Please circle one)*

Very confident *Confident* *Not very confident* *Not confident at all*

5. How confident do you feel that you will be able to use this in the future? *(Please circle one)*

Very confident *Confident* *Not very confident* *Not confident at all*

PLEASE TURN OVER THE PAGE FOR REMAINING QUESTIONS

IWOTCH Participant Feedback Form V1.0 09Oct18. IRAS reference: 199154

This project was funded by the National Institute for Health Research, Health Technology Assessment (project number 14/224/04).
The views and opinions expressed therein are those of the authors and do not necessarily reflect those of the HTA, NIHR, NHS or the Department of Health.

Funded by NIHR

Group venue: ______________________________

Date: ______________________________

6. Overall were the facilitators: *(Please circle one)*

Very good *Good* *Satisfactory* *Poor*

7. Overall were the handouts: *(Please circle one)*

Very good *Good* *Satisfactory* *Poor*

8. How did you find the face to face meeting with the nurse? *(Please circle one)*

Very useful *Useful* *Not very useful* *Not useful at all*

9. How did you find the telephone calls with the nurse? *(Please circle one)*

Very useful *Useful* *Not very useful* *Not useful at all*

10. Overall how useful did you find the whole course? *(Please circle one)*

Very useful *Useful* *Not very useful* *Not useful at all*

11. Is there anything else you would like to say?

Thank you.

Please return in the stamped addressed envelope

IWOTCH Participant Feedback Form V1.0 09Oct18. IRAS reference: 199154

This project was funded by the National Institute for Health Research, Health Technology Assessment (project number 14/224/04).
The views and opinions expressed therein are those of the authors and do not necessarily reflect those of the HTA, NIHR, NHS or the Department of Health.

Funded by NIHR
