# Supplementary material for: Process evaluation protocol for the I-WOTCH study: an opioid tapering support programme for people with chronic non-malignant pain
Source: BMJ Open. 2019 Oct 10;9(10):e028998. doi: 10.1136/bmjopen-2019-028998 (PMC6797361; doi:10.1136/bmjopen-2019-028998)
Supplement: Supplementary data [file bmjopen-2019-028998supp002.pdf]

DAY 1 I-WOTCH Fidelity checklists

Group ID: **Date of group session:**

**Day 1: Living with and dealing with pain**	**Date listened**	**Occurred/ Did not**	**Adherence score**	**Competence score**	**Comments**
Session 1 Introduction 15mins					
***Session 2**<br>**Pain information approx. 45mins**					
***Session 3**<br>**Painkiller information and opioid education approx. 45mins**					
Break					
***Session 4**<br>**Acceptance: John's story 20mins**					
Session 5 Attention control and distraction 10mins					
Session 6 Distraction activity – rose drawing 20mins					
Lunch					
***Session 7**<br>**Good days, Bad days when is pain bearable and when is it not? 40mins**					
***Session 8**<br>**The pain cycle unhelpful emotions and behaviours 40mins**					
Break					
Session 9 Posture					
Session 10 Relaxation and Breathing					
Session 11 Summary of the day					
**Total scores**					

Comments: *e.g. session not recorded so subsequent group same session listened to instead*
