# Supplementary material for: Process evaluation protocol for the I-WOTCH study: an opioid tapering support programme for people with chronic non-malignant pain
Source: BMJ Open. 2019 Oct 10;9(10):e028998. doi: 10.1136/bmjopen-2019-028998 (PMC6797361; doi:10.1136/bmjopen-2019-028998)
Supplement: Supplementary data [file bmjopen-2019-028998supp003.pdf]

I-WOTCH course code: _____ Reviewer: __________________________ Review date: ____________

Day 1 /Session 2 /Title: Pain Information 30mins

Adherence: of the delivery as per protocol

No.	Item	Adherence	Comments
Intro	Did the facilitator(s) introduce the session?	Yes (2) Partially (1) No (0)	
Step 1	Did the facilitator(s) play the DVD of the biomedical explanation about acute and chronic pain?	Yes (2) Partially (1) No (0)	
	Did the facilitator(s) ask the group Q1 and discuss, “What do you think about this explanation of pain? Is it missing anything?”	Yes (2) Partially (1) No (0)	
Step 2	Did the facilitator(s) present the bio-psycho-social explanation of pain?	Yes (2) Partially (1) No (0)	
	Did the facilitator(s) ask the group Q2 and discuss, “What do you think about this explanation of pain?”	Yes (2) Partially (1) No (0)	
Step 3	Did the facilitator(s) play the DVD of Experiences of living with opioid- treated long term pain?	Yes (2) Partially (1) No (0)	
	Did the facilitator(s) ask the group Q3 and discuss, “What do you think about Caroline’s description of living with opioid-treated long-term pain?”	Yes (2) Partially (1) No (0)	
Summary	Did the facilitator(s) consolidate/embed the group’s learning at the end of the session? *e.g. reading the summary, putting the session in context*	Yes (2) Partially (1) No (0)	
	Total adherence score (max 16)		
	Percentage adherence score (Total adherence score */16x100)		

Instructions:

When at all possible please rate as ‘Yes’ or ‘No’ If ‘partially’ then write reason in comments box
Questions need not be verbatim (unless specified) as long as content of session is covered.

I-WOTCH course code: _____ **Reviewer: __________________________** **Review date: ____________**

**Comments:** For use if sessions; go off track, include items which are not on checklist, contain surprising unforeseen aspects or the facilitation wasn't covered as intended. Also if there was no opportunity to demonstrate the skill listed.

I-WOTCH course code: _____ Reviewer: _________________________ Review date: ____________

Day 1 /Session 2 /Title: Pain Information and Opioid Education

Competence of the quality of delivery or 'skill' of the facilitators

	Item	Competence measure	Comments (*use box below to expand)*
1	Did the facilitator(s) create opportunities for discussion *e.g. did they; encourage individuals to participate, ask open questions, give enough time for the group to answer (rather than answer their own questions)*	Evident (2)<br>Partially evident (1)<br>Not evident (0)<br>Did not happen in this session (N/A)	
2	Did the facilitator encourage active participation across group members? *e.g. did they encourage quieter members and manage dominant members?*	Evident (2)<br>Partially evident (1)<br>Not evident (0)<br>Did not happen in this session (N/A)	
3	Did the facilitator(s) encourage individual disclosure? *e.g. did they ask different group members to comment or encourage the group to explore issues further (either individually or as a group)?*	Evident (2)<br>Partially evident (1)<br>Not evident (0)<br>Did not happen in this session (N/A)	
4	Did the facilitator(s) validate participants' disclosures? *e.g. Do other people find this/think that? I know how you feel. Sometimes people may feel differently about things.*	Evident (2)<br>Partially evident (1)<br>Not evident (0)<br>Did not happen in this session (N/A)	
5	Did the facilitator(s) give encouraging feedback on participants reported behaviours? *e.g. Did they give appraisal 'that's really good' or 'that's really good but I wonder if…'*	Evident (2)<br>Partially evident (1)<br>Not evident (0)<br>Did not happen in this session (N/A)	
6	Did the facilitator(s) foster a positive group climate? *e.g. did they; use humour, say positive things about people 'that's a helpful comment' 'thank you for sharing that'*	Evident (2)<br>Partially evident (1)<br>Not evident (0)<br>Did not happen in this session (N/A)	
7	Did the facilitator acknowledge and respond appropriately to admissions or statements of low self-efficacy? e.*g. 'yes this can be difficult but…' ideas or examples offered of how this may be done.*	Evident (2)<br>Partially evident (1)<br>Not evident (0)<br>Did not happen in this session (N/A)	
8	Did the facilitator respond appropriately to disclosures of negative events?	Evident (2)<br>Partially evident (1)<br>Not evident (0)<br>Did not happen in this session (N/A)	

I-WOTCH course code: _____ **Reviewer: __________________________** **Review date: ____________**

	Total competence score (max 16)		
	Percentage competence score		

Comments: For use if sessions; go off track, include items which are not on checklist, contain surprising unforeseen aspects or the facilitation wasn't covered as intended. Also if there was no opportunity to demonstrate the skill listed.
