# Supplementary material for: Process evaluation protocol for the I-WOTCH study: an opioid tapering support programme for people with chronic non-malignant pain
Source: BMJ Open. 2019 Oct 10;9(10):e028998. doi: 10.1136/bmjopen-2019-028998 (PMC6797361; doi:10.1136/bmjopen-2019-028998)
Supplement: Author's manuscript [file bmjopen-2019-028998.draft_revisions.pdf]

BMJ Open is committed to open peer review. As part of this commitment we make the peer review history of every article we publish publicly available.

When an article is published we post the peer reviewers' comments and the authors' responses online. We also post the versions of the paper that were used during peer review. These are the versions that the peer review comments apply to.

The versions of the paper that follow are the versions that were submitted during the peer review process. They are not the versions of record or the final published versions. They should not be cited or distributed as the published version of this manuscript.

BMJ Open is an open access journal and the full, final, typeset and author-corrected version of record of the manuscript is available on our site with no access controls, subscription charges or pay-per-view fees (http://bmjopen.bmj.com).

If you have any questions on BMJ Open's open peer review process please email info.bmjopen@bmj.com

BMJ Open

PROCESS EVALUATION PROTOCOL FOR THE IWOTCH STUDY: AN OPIOID TAPERING SUPPORT PROGRAMME FOR PEOPLE WITH CHRONIC NON-MALIGNANT PAIN.

Journal:	*BMJ Open*
Manuscript ID	bmjopen-2019-028998
Article Type:	Protocol
Date Submitted by the Author:	07-Jan-2019

Complete List of Authors:	Nichols, Vivien; University of Warwick, Warwick Clinical Trials Unit<br>Abraham, Charles; University of Melbourne, Faculty of Medicine, Dentistry and Health Sciences<br>Eldabe, Sam; The James Cook University Hospital<br>Sandhu, Harbinder; University of Warwick Warwick Medical School, Clinical Trials Unit<br>Underwood, Martin; Warwick University, Warwick Medical School<br>Seers, Kate; University of Warwick, Warwick Research in Nursing, Warwick Medical School

Keywords:	Process Evaluation, Opioid, Protocol, Chronic Non-Malignant pain, Tapering

SCHOLARONE™
Manuscripts

PROCESS EVALUATION PROTOCOL FOR THE IWOTCH STUDY: AN OPIOID TAPERING SUPPORT PROGRAMME FOR PEOPLE WITH CHRONIC NON-MALIGNANT PAIN.

Authors: Vivien Nichols*, Charles Abraham, Sam Eldabe, Harbinder Sandhu, Martin Underwood and Kate Seers on behalf of the IWOTCH team (see acknowledgements)

*Corresponding author

Vivien P Nichols, Warwick Clinical Trials Unit, Division of Health Sciences,

Warwick Medical School, University of Warwick, Coventry CV4 7AL, UK

(+44) 0247657465

V.P.Nichols@warwick.ac.uk

Charles Abraham, School of Psychological Sciences, Faculty of Medicine, Dentistry and Health Sciences,

University of Melbourne, Victoria 3010, Australia

charles.abraham@unimelb.edu.au

Sam Eldabe, Department of Pain Management, The James Cook University Hospital

Marton Road, Middlesbrough TS4 3BW

seldabe@nhs.net

Harbinder Sandhu, Warwick Clinical Trials Unit, Division of Health Sciences,

Warwick Medical School, University of Warwick, Coventry CV4 7AL, UK

Harbinder.K.Sandhu@warwick.ac.uk

Martin Underwood, Warwick Clinical Trials Unit, Division of Health Sciences,

For peer review only

For peer review only - http://bmjopen.bmj.com/site/about/guidelines.xhtml

Warwick Medical School, University of Warwick, Coventry CV4 7AL, UK and University Hospitals Coventry & Warwickshire NHS Trust, University Hospital, Clifford Bridge Road Coventry, CV2 2DX

M.Underwood@warwick.ac.uk

Kate Seers, Warwick Research in Nursing, Warwick Medical School, University of Warwick, Coventry, CV4 7AL, UK.

Kate.Seers@warwick.ac.uk

For peer review only

Word Count: 2,176

Key words: Process Evaluation, Opioids, Protocol, Chronic Non-Malignant Pain, Tapering

Abstract

Introduction

The Improving the Wellbeing of people with Opioid Treated CHronic pain (I-WOTCH) randomised controlled trial uses a multi component self-management intervention to help people to taper their opioid use. This approach is not widely used and its efficacy is unknown. A process evaluation alongside the trial will help to assess how the intervention was delivered looking at the dose of intervention received and the fidelity of the delivery. We will explore how the intervention may have brought about change through the experiences of the participants receiving and the staff delivering the intervention and whether there were contextual factors involved.

Methods and analysis

A mixed methods process evaluation will assess how the processes of the IWOTCH intervention fared and whether these affected the outcomes. We will collect quantitative data e.g. group attendance analysed with statistical methods. Qualitative data e.g. from interviews and feedback forms will be analysed using framework analysis. We will use a 'following a thread' and a mixed methods matrix for the final integrated analysis.

Ethics and dissemination

The IWOTCH trial and process evaluation have been granted full ethics approval by Yorkshire & The Humber - South Yorkshire Research Ethics Committee on September 13th, 2016 (16/YH/0325). All data has been collected in accordance with data protection guidelines. Participants provide written informed consent for the main trial and all interviewees provide additional written informed consent. The results of the process evaluation will be published and presented at conferences.

For peer review only - http://bmjopen.bmj.com/site/about/guidelines.xhtml

Trial registration:

This trial is registered with an International Standard Randomised Controlled Trial Number (ISRCTN) Register. ISRCTN number: 49470934 (06 Feb 2017).

Article Summary

- Little is known about whether a multi component self-management intervention can help people to taper their opioid use.
- Process evaluation allows for exploration into how a study was implemented, how processes fared and whether these were carried out as intended.
- Qualitative interviews give insight into how people experienced the study both from those delivering and receiving the intervention.
- Using a mixed methods approach will enable us to explore lines of argument across the trial data.

Introduction

The I-WOTCH study

The I-WOTCH study (Improving the Wellbeing of people with Opioid Treated CHronic pain) is a randomised controlled trial testing the effectiveness and cost effectiveness of a patient-centred, multicomponent self-management intervention targeting withdrawal of strong opioids among those living with chronic non-malignant pain. Primary outcomes are activities of daily living measured by the Patient-Reported Outcomes Measurement Information System (PROMIS) Pain Interference Short Form (8A) (PROMIS-PI-SF-8A) and opioid dose reduction measured as morphine daily dose equivalent at 12 months follow up. Trial participants are identified from general practice records, using electronic searches and approached by letter. They are randomised into the control group who receive the 'My Opioid Manager' self-help guide and a relaxation CD or to the intervention group who are invited to attend three days of group activities, two one-to-one sessions with a clinical facilitator (usually a nurse) after day two and up to

For peer review only - http://bmjopen.bmj.com/site/about/guidelines.xhtml

two follow- up telephone calls between day three and the last one-to-one session. I-WOTCH is a multisite trial, requiring standardisation of training and delivery. The intervention is a complex and multicomponent, including educational and behaviour change components. Any changes in medication are discussed with, and if appropriate, any additional medications are prescribed by their GP. Outcomes will be assessed at four time points, baseline, four, eight and twelve months. The I-WOTCH protocol paper gives greater detail about the study.(1)

Preliminary Work

We did a formative process evaluation as part of an intervention pilot study which found that the randomisation and the control arm seemed acceptable and the paperwork was not reported to be burdensome. The delivery of groups and intervention attendance showed that group delivery was feasible, numbers were lower than expected and strategies were put in place to improve this in the main study. Once people attended day one attendance was good; and those who could not attend the first session (most often due to work commitments or poor health) were offered a different future group. Observation of one group reported good group engagement and facilitation of group content and discussions were well received by the participants attending. Feedback from participants was positive about the course, the most useful aspects being the gaining of new knowledge about opioids and pain within a supportive environment. Participants also said that they found the components of the course which helped them to change their thoughts and attitudes to their pain useful. Things which they would change about the group sessions centred on practical considerations such as the comfort of seating and better sound equipment. This feasibility work helped us to develop our logic model and specific components of the main trial process evaluation such; as our interview topic guides and fidelity paperwork to assess adherence and competence.

Process evaluation

This paper describes a process evaluation that is being conducted as an integral part of the I-WOTCH trial. We are doing a mixed methods process evaluation based on MRC guidance, to better understand how the intervention works.(2) Key foci of evaluation, as described by Steckler and Linnan are; context, (contextual factors which may affect the implementation), fidelity (whether the intervention delivered as designed) dose

For peer review only - http://bmjopen.bmj.com/site/about/guidelines.xhtml

delivered (the amount of the intervention delivered), dose received (the amount of the intervention received by participants) and reach (who are the participants and where do they come from).(3) We will assess fidelity, ascertaining whether the trial processes were conducted as per protocol so minimising possible type three errors e.g. when the outcomes of a study do not take into account an inadequate implementation of an intervention.(4) We will identify any delivery which deviates from the original design because these may be important when interpreting the trial results. We will also investigate (i) how the contexts (e.g., different sites) of implementation affected delivery, (ii) how implementation of the intervention was managed and (iii) whether the hypothesised change mechanisms operated as expected. These data can inform replication, development and integration of interventions within routine practice so assisting researchers, commissioners and practitioners.

The process evaluation team (KS, VN and CA) will work independently of the main trial team during the data collection phase to avoid contamination of trial processes. Findings from the process evaluation may provide insights which could enhance interpretation of the trial results.

Aims:

In summary, the aims of this process evaluation are to investigate:-

1) Experiences of the intervention including enablers of, and barriers to, the intervention facilitating change among participants.
2) Intervention implementation, exploring dose of the intervention delivered and received and the fidelity of delivery.
3) Change mechanisms assessing whether hypothesised change occurred.
4) Contextual issues that may affect the outcome or running of the study and/or intervention.

Methods

The aim of the IWOTCH intervention is to improve participants' quality of life and reduce their use of opioid drugs. We have developed a logic model specific to this intervention guided by Intervention Mapping principles.(5) See figure 1. We have also considered items from a checklist of key features of any group

For peer review only - http://bmjopen.bmj.com/site/about/guidelines.xhtml

intervention that need to be reported to ensure replication enabling us to identify a series of characteristics to investigate.(6) The intervention includes educational, psychological and behavioural components designed to effect change. Thus the change mechanisms can be conceptualised in terms of the Information, Motivation and Behaviour skills (IMB) model developed by Fisher and Fisher (1992),(7) hypothesising that the intervention will (i) provide useful new information concerning the effects of opioids, (ii) motivate participants to reduce their reliance on opioids and (iii) provide them with new skills to facilitate non-opioid pain control, see figure 2.

We will use a mixed methods approach, using quantitative data collected by the trial team as well as qualitative data collection methods outlined in the sections below which map into our aims.

1) Experiences of the intervention including enablers of, and barriers to, the intervention facilitating change among participants.

Qualitative data will include interviews with participants and those delivering the intervention. Participant feedback forms include qualitative and quantitative data from open and satisfaction questions respectively.

Table 1 Interview and participant feedback data

Key Components	Source of Data	Type of Data
Experiences of participants.<br>Interview topics including:<br>• Responses to receiving the intervention or control<br>• How they felt they were able to use it<br>• How easy or difficult it was to use?<br>• Were some components more challenging than others?<br>• Specific barriers and enablers.<br>• Experience of being in a group. (intervention only)	Participants	Interview recordings and transcripts
Participant feedback forms	Intervention participant forms	Feedback form questions
Experiences of delivering the intervention	Intervention delivery staff (clinical facilitator and lay person with	Interview recordings and transcripts

For peer review only - http://bmjopen.bmj.com/site/about/guidelines.xhtml

	chronic pain or allied health professional)	

Interviews

Participants will be interviewed after their final follow up (at 12 months) to minimise possible effects of the interview on the trial findings. The interviews will be semi-structured and held in a convenient local venue to the participant. We will interview up to 20 intervention participants and 20 who were allocated to the control group. These will be purposively sampled to ensure a range of age, gender, location and opioid reduction experience across the two trial arms.

Participants will have agreed to be contacted about a possible interview on their initial trial consent form and, after receiving the 12-month follow-up questionnaires, our sample will be sent a patient information leaflet inviting them to take part in a face-to face interview about their experiences of being part of the study. After a week a researcher will contact them by phone to answer any questions and if agreeable book an appointment. A separate informed consent process will be completed at the beginning of each interview.

We will interview up to 20 trained intervention staff across different geographic areas. These include clinical facilitators, (usually a nurse) whose role was to; facilitate groups, see participants for their one-to-one appointments and give them telephone support as required. We will also interview the other group facilitators, either a lay person with experience of opioid use and tapering or an allied health practitioner with an interest in chronic pain conditions. Approach will be by an invitation letter with an information leaflet and consent will be taken before the interview. Interviews will be semi structured using a topic guide, and take place after the interventions have been completed.

All interviews will be audio-recorded and transcribed verbatim when all identifiable data will be removed. They will then be checked for accuracy by the interview researcher. Audio recordings will be held in a digitally secure environment with restricted access.

We will analyse the interviews using both thematic analysis and framework analysis.(8, 9) Transcripts will be analyzed using the six steps outlined by Braun and Clarke (2006). After thorough familiarization with the data through listening to all recordings, and reading and re-reading the transcripts, five interviews will be

For peer review only - http://bmjopen.bmj.com/site/about/guidelines.xhtml

analyzed by coding themes related to the research questions. The emerging lower-level codes will then be grouped into higher-level themes, related to the research questions. All transcripts will then be coded using the hierarchical coding framework, paying attention to any new themes and deviant cases. We will review data related to each code and theme, check and re-coded if necessary, and define themes. Throughout the analysis the analysis team will make reflective analytic memos and hold regular discussion meetings. We will use NVivo qualitative data analysis software; (QSR International Pty Ltd.) to organise the data.

Feedback forms

Feedback forms will be given to intervention participants after their last group or at their second one-to-one session. These forms are anonymous and will be sent back to the Warwick Clinical Trials Unit in a stamped addressed envelope to ensure anonymity. These forms contain quantitative satisfaction questions which will be analysed statistically and open questions which will be analysed using thematic analysis. See appendix 1

2) Intervention implementation exploring dose of the intervention delivered and received, and the fidelity of delivery

We will note the uptake and attendance of the different components of the intervention, to allow assessment of the intervention dose delivered and received, see table 2.

Table 2 Quantitative Data on Dose Delivered and Received

Key Components	Potential Source of Data	Type of Data
Intervention groups	Trial data	Groups run, location and dates
Numbers attending each component of the three intervention days	Trial data	Attendance sheets per session
Uptake of the one-to-one sessions	Trial data<br>Intervention staff	Intervention trial log<br>Staff interviews
Uptake of the telephone follow up telephone calls.	Trial data<br>Intervention staff	Intervention trial log<br>Staff interviews

Fidelity of Intervention Delivery

Fidelity will be assessed using audio recordings of the group and one-to-one sessions. See table 3

Table 3 Fidelity of Intervention Delivery

Key Components	Source of Data	Type of Data

For peer review only - http://bmjopen.bmj.com/site/about/guidelines.xhtml

Assess fidelity of group sessions<br>• Adherence<br>• Competence	Audio recordings of all sessions	Adherence and competence ratings with researcher notes from a selection of sessions
One to one sessions<br>To understand the issues discussed	Audio recordings of all sessions	Adherence and competence ratings with researcher notes from a selection of first and second interviews

The I-WOTCH protocol has a target of 468 participants (234 of whom will be allocated to the intervention) by running 24 groups. All intervention sessions and one to one consultations will be audio recorded. A statistician using random number generator will choose randomly; nine Day 1 sessions, nine Day 2 sessions and nine Day 3 sessions from early, middle and late stages of the study this will ensure we listen to approximately 10% of group sessions. Through extensive discussions with the team who developed the intervention we decided to pre-specify those sessions which were considered by the team to be key to promoting behaviour change and contain either educational or discussion items. Other sessions which are more practical in nature (e.g. origami for distraction) will be checked to see if they took place but will not be rated for facilitator adherence and competence. See table 4.

Table 4 Course programme with sessions identified for fidelity

**DAY 1**
Session 1 Introduction
***Session 2 Pain information**
***Session 3 Painkiller information and opioid education**
***Session 4 Acceptance: John's story**
Session 5 Attention Control and distraction
Session 6 Distraction activity – rose drawing
***Session 7 Good days, Bad days when is pain bearable and when is it not?**
***Session 8 The pain cycle unhelpful emotions and behaviours**
Session 9 Posture
Session 10 Relaxation and Breathing
Session 11 Summary of the day
**DAY 2**
Session 12 Reflections from day 1
***Session 13 Stress-busting – prioritising what's important, action planning, goal setting and pacing**
***Session 14 Withdrawal symptoms, case studies (Opioid Education 2)**
Session 15 Distraction activity- origami
***Session 16 Identifying and overcoming Barriers to change Part 1 – recognising unhelpful thinking**
***Identifying and overcoming Barriers to change Part 2– reframing negatives to positives**
Session 17 Mindful attention control
Session 18 Balance and introduction to stretch
Session 19 Summary of the Day
**DAY 3**
Session 20 Reflections from day 2 and previous week
***Session 21 Anger, irritability and frustration**
***Session 22 Relationships Part 1 Getting the most from your healthcare team**
Session 22 Part 2 Relationships Part 2 Listening skills
***Session 23 Managing setbacks and non-drug management techniques**

For peer review only - http://bmjopen.bmj.com/site/about/guidelines.xhtml

For peer review only

Session 24 Distraction activity – mindfulness colouring		
Session 25 Stretching muscles that commonly get tight		
Session 26 Mindfulness of thoughts and Senses		
Session 27 Summary of Day 3		
Session 28 Summary of the course		
Legend	***Educational and/or self-management regarding pain or opioid use**	Practical, reflection or summarising sessions
Day 1	**2, 3, 4, 7, 8**	1,5,6,9,10,11
Day 2	**13,14,16**	12,15,17,18,19
Day 3	**21,22 part 1,23,**	20,22 part 2, 24,25,26,27,28

To assess the fidelity of the intervention we will assess two aspects of intervention delivery; adherence to the intervention manual and competency of the facilitators. A member of the process evaluation team will listen to a random selection of 10% of the group sessions and one-to-one nurse consultations. They will score adherence and competence using a specially devised checklist based upon components specified in the manual items and training protocol for intervention facilitators. See appendices 2 and 3 for examples. We will also rate one of the first or second one-to-one nurse consultations per group N=24. We will double rate 10% of these group and one-to-one sessions by a second member of the team to assess inter-rater reliability and to ensure rigour. Percentage scores will be given for adherence and competence per session and the findings analysed using standard statistical methods.

3) Change Mechanisms assessing whether hypothesised change occurred.

We will administer self-report questionnaires to track possible change mechanisms. Specifically we will assess participants' (i) motivation to reduce opioid use before and after the intervention, (ii) expectations of success in opioid reduction and (ii) confidence (or self-efficacy (10)) in relation to opioid reduction prior to receiving the intervention and (iv) perceived intervention efficacy before and after participation, see table 5. These data will be analysed using standard statistical methods, including t tests and ANCOVAs.

For peer review only - http://bmjopen.bmj.com/site/about/guidelines.xhtml

Table 5 Motivation Expectations, Self-Efficacy and Perceived Intervention Efficacy Questions

**Baseline Motivation** (baseline and follow up)<br>**I want to reduce my opioid use**<br>(Not at all, by a little, by half, so I only use a little, so I use no opioids)<br><br>**Baseline Expectation** (baseline only)<br>**I expect that, in 4 months' time, I will have reduced my opioid use:**<br>(Not at all, by a little, by half, so I only use a little, so I use no opioids)<br><br>**Baseline Self-Efficacy** (baseline only)<br>**I am confident I could reduce my opioid use a lot over 4 months**<br>(Not at all confident, somewhat confident, fairly confident, strongly confident, completely confident)<br><br>**Perceived Intervention Efficacy** (baseline and follow up)<br>**Baseline**<br>**I feel that involvement in this study can help me to reduce my opioid use**<br>Not at all, by a little, by half, so I only use a little, so I use no opioids<br><br>**Follow up**<br>**I feel that involvement in this study has helped me to reduce my opioid use**<br>Not at all, by a little, by half, so I only use a little, so I use no opioids

4) Contextual issues that may affect the outcome or running of the study and/or intervention

Contextual factors may be found in the data collected above may influence change and outcomes. We will explore this as the need arises from the data as it may be a 'thread to follow' (see paragraph below) or an integral part of a section of the analysis.

Mixed Methods Analysis

Data from quantitative and qualitative findings will be integrated as outlined by O'Cathain et al.(11) We will use both 'following a thread' which involves selecting a question or component from one aspect of the findings and following across, and 'mixed methods matrix' where, for example, responses on quantitative scales can be compared to interview transcript, and data on each case can be concisely stated and recorded on a matrix.(11)

Trial Status

The I-WOTCH study began recruitment in May 2017 and anticipate groups will be running into February 2019, data collection will be completed around February 2020 and they expect the final report for the funders will be submitted mid-2020.

For peer review only - http://bmjopen.bmj.com/site/about/guidelines.xhtml

Ethics and Dissemination

We intend to publish the process evaluation findings in peer reviewed journals and details of the main trial ethics and dissemination are outlined in the main trial protocol.(1)

The IWOTCH trial and process evaluation have been granted full ethics approval by Yorkshire & The Humber - South Yorkshire Research Ethics Committee on September 13th, 2016 (16/YH/0325).

References

1. Sandhu H, Abraham C, Alleyne S, et al. Testing a support programme for opioid tapering for people with chronic non-malignant pain: The I-WOTCH Study protocol submitted to BMJopen2018.
2. Moore GF, Audrey S, Barker M, et al. Process evaluation of complex interventions: Medical Research Council guidance. BMJ2015;350:h1258.
3. Steckler A, Linnan L. Process Evaluation for Public health Interventions and research. San Fransisco: Jossey-Bass; 2002.
4. Basch CE, Sliepcevich EM, Gold RS, et al. Avoiding type III errors in health education program evaluations: a case study. Health Educ Q1985;12(4):315-31.
5. Bartholomew Eldredge LK, Markham CM, Ruiter RAC, et al. Planning health promotion programs: Jossey-Bass Inc.; 2016 2016.
6. Borek AJ, Abraham C, Smith JR, et al. A checklist to improve reporting of group-based behaviour-change interventions. BMC Public Health2015;15:963.
7. Fisher JDF, W.A. The Information-Motivation-Behavioural Skills Model. In: DiClemente R, Crosby R, Kegler M, editors. Emerging theories in health promotion practice and research: Strategies for improving public health. San Fransisco: Jossey-Bass; 2002. p. 40-7.
8. Ritchie J, Spencer L. Qualitative data analysis for applied policy research. The qualitative researcher's companion2002;573(2002):305-29.
9. Braun V, Clarke V. Using thematic analysis in psychology. Qualitative Research in Psychology2006;3(2):77-101.
10. Bandura A. Self-efficacy: toward a unifying theory of behavioral change. Psychol Rev1977;84(2):191-215.
11. O'Cathain A, Murphy E, Nicholl J. Three techniques for integrating data in mixed methods studies. BMJ (Online)2010;341(7783):1147-50.

Authors' contributions

KS, MU and HS conceived the original study design. VN, KS and CA have developed the study design, and plan for data collection and analysis. All authors have provided critical revisions to the manuscript and approved the final manuscript.

For peer review only - http://bmjopen.bmj.com/site/about/guidelines.xhtml

Funding

This project is funded by the National Institute for Health Research (NIHR), Health Technology Assessment (HTA) (project number 14/224/04). The views and opinions expressed therein are those of the authors and do not necessarily reflect those of the HTA, NIHR, NHS or the Department of Health.

Competing interests statement.

SE is investigator on a number of NIHR and industry sponsored studies. He received travel expenses for speaking at conferences from the professional organisations. SE consults for Medtronic, Abbott, Boston Scientific and Mainstay Medical, none in relation to opioids. SE is chair of the BPS Science and Research Committee. SE is deputy Chair of the NIHR CRN Anaesthesia Pain and Perioperative Medicine National Specialty Group. SE's department has received fellowship funding from Medtronic as well as nurse funding from Abbott.

HS is director of Health Psychology Services Ltd, providing psychological services for a range of health related conditions.

MU was Chair of the NICE accreditation advisory committee until March 2017 for which he received a fee. He is chief investigator or co-investigator on multiple previous and current research grants from the UK National Institute for Health Research, Arthritis Research UK and is a co-investigator on grants funded by the Australian NHMRC. He is an NIHR Senior Investigator. He has received travel expenses for speaking at conferences from the professional organisations hosting the conferences. He is a director and shareholder of Clinvivo Ltd that provides electronic data collection for health services research. He is part of an academic partnership with Serco Ltd related to return to work initiatives. He is a co-investigator on a study receiving support in kind from Orthospace Ltd. He is an editor of the NIHR journal series, and a member of the NIHR Journal Editors Group, for which he receives a fee.

KS received grant funding as PI and CoI from NIHR for other projects and was on the NIHR HS&DR Funding Board until January 2018.

For peer review only

For peer review only - http://bmjopen.bmj.com/site/about/guidelines.xhtml

Acknowledgements

This manuscript was written on behalf of the I-WOTCH team:

Sharisse Alleyne, University of Warwick
Shyam Balasubramanian, Department of Anaesthesia & Pain Medicine University Hospitals Coventry and Warwickshire NHS Trust
Lauren Betteley, University of Warwick
Katie Booth, University of Warwick
Dawn Carnes, Barts & The London Queen Mary's School of Medicine & Dentistry
Andrea Dompieri Furlan, Toronto Rehabilitation Institute, University Health Network, Canada
Kirstie Haywood, University of Warwick
Ranjit Lall, University of Warwick
Andrea Manca, The University of York
Dipesh Mistry, University of Warwick
Jennifer Noyes, The James Cook University Hospital
Anisur Rahman, University College London
Jane Shaw, The James Cook University Hospital
Nicole Kit Yee Tang, University of Warwick
Stephanie Taylor, Barts & The London Queen Mary's School of Medicine & Dentistry
Colin Tysall, University/User Teaching and Research Action Partnership, University of Warwick
Emma Withers, University of Warwick
Martin Underwood, University of Warwick
Cynthia Paola Iglesis Urrutia, The University of York

For peer review only - http://bmjopen.bmj.com/site/about/guidelines.xhtml

Figure 1 Logic model

The problem	Intervention Aims	Intervention	Theory and Guidance	Interim Targets	Desired Outcomes
People with chronic non-malignant pain are taking opioids, which have side effects and are not effective in the long term.	To test the effectiveness and cost effectiveness of a patient-centred multicomponent self-management intervention targeting withdrawal of strong opioids on activities of daily living for people living with chronic non-malignant pain	**Manualised Intervention Delivery**<br>**Core pain management topics:**<br>• Acute versus Chronic pain<br>• Acceptance<br>• Attention Control and distraction<br>• the pain cycle<br>• Posture and movement advice<br>• Relaxation techniques<br>• Stress busting for health action planning, problem solving, pacing, SMART goal setting<br>• identifying and overcoming barriers to change<br>• Mindfulness<br>• Anger, irritability and frustration<br>• Communication Skills<br><br>**Core opioid specific topics:**<br>• The rationale of prescribing in chronic pain<br>• Opioid induced tolerance and need for dose escalation<br>• Evidence of usefulness of opioids short and long term<br>• Side effects of opioids short term and long term<br>• Case studies of successful discontinued opioid therapy<br>• Opioid withdrawal symptoms<br>• Advantages of slow supervised tapering<br>• Symptom management during tapering<br>• Pain control after opioids	Theory of Planned Behaviour<br><br>Social Cognitive Theory<br><br>Information Motivation and Behavioural (IMB model) skills<br><br>Patient Centred Communication<br><br>Motivational Interviewing	**Staff Training**<br>To facilitate groups, deliver individual tapering consultations and telephone support in an inclusive and non-judgemental manner<br>**Individual participant changes:**<br>a Knowledge of: opioids, withdrawal effects, chronic pain<br>b Fostering change: self-validation, legitimising pain, normalising expectations<br>c Motivation to change by: Improved self-efficacy, effective tapering<br>d Skills:<br>• General Self-Regulation<br>*Psychological skills*<br>*Identify reasons for negative emotions (anger /frustration /irritable)*<br>*Identify problems and solutions, barriers to change*<br>*Recognise errors in thinking/automatic thoughts*<br>*Goal setting, goal review*<br>*Physical skills*<br>*Promote body awareness, posture*<br>*Reduce muscle tension*<br>*Body awareness and core strength*<br>*Relaxation-contract relax*<br>• Pain Self-Regulation<br>Understand that pain and mood are linked – when is pain bearable and when not bearable.<br>Understanding of pain cycle, unhelpful emotions and behaviours<br>Using mind to relieve pain does not mean pain in mind<br>Distraction whilst relaxed<br>Focus mind away from pain<br>Mindfulness for pain<br>Managing flare ups<br>Need for stretching<br>• Communication Skills<br>How to communicate with General Practitioners (GPs) and Health Care Professionals (HCPs)<br>Listening skills - Active and giving feedback in communication-reward for help.	**Primary outcomes:**<br>Patient-Reported Outcomes Measurement Information System (PROMIS) Pain Interference Short Form (8A)(PROMIS-PI-SF-8A)<br><br>Daily morphine equivalent opioid dose

For peer review only

Figure 2. The Information-Motivation-Behavioral Skills Model (Fisher & Fisher, 1992)

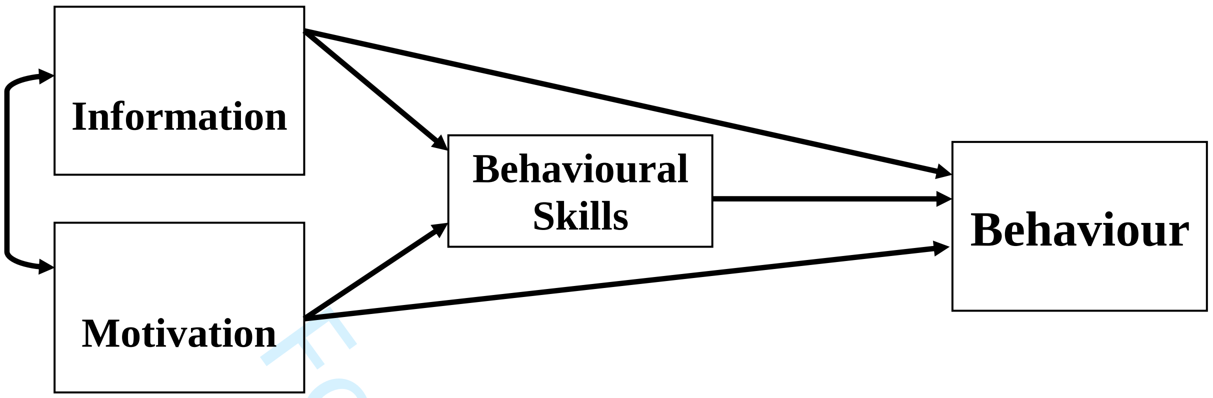

For peer review only

I-WOTCH IMPROVING THE WELLBEING OF PEOPLE WITH OPIOID TREATED CHRONIC PAIN

Group venue: ________________________________

Date: ________________________________

I-WOTCH Feedback form

Thank you for attending the I-WOTCH course. Please complete the feedback and short questionnaire below. This information will be used to evaluate the support programme. Please note that these forms are anonymous and will be sent back to the study team.

1. Were the aims of the course made clear? Please circle one

Yes *No* *Don't know*

2. What were the three most useful things on this course?

1)………

2)………

3)………

3. What three things would you suggest to make this course better for future participants?

1) ………

2) ………

3) ………

4. How confident do you feel that the course content will help you personally? *(Please circle one)*

Very confident *Confident* *Not very confident* *Not confident at all*

5. How confident do you feel that you will be able to use this in the future? *(Please circle one)*

Very confident *Confident* *Not very confident* *Not confident at all*

PLEASE TURN OVER THE PAGE FOR REMAINING QUESTIONS

This project was funded by the National Institute for Health Research, Health Technology Assessment (project number 14/224/04).
The views and opinions expressed therein are those of the authors and do not necessarily reflect those of the HTA, NIHR, NHS or the Department of Health.

Funded by NIHR

For peer review only - http://bmjopen.bmj.com/site/about/guidelines.xhtml

For peer review only

Group venue: ______________________________

Date: ______________________________

6. Overall were the facilitators: *(Please circle one)*

Very good *Good* *Satisfactory* *Poor*

7. Overall were the handouts: *(Please circle one)*

Very good *Good* *Satisfactory* *Poor*

8. How did you find the face to face meeting with the nurse? *(Please circle one)*

Very useful *Useful* *Not very useful* *Not useful at all*

9. How did you find the telephone calls with the nurse? *(Please circle one)*

Very useful *Useful* *Not very useful* *Not useful at all*

10. Overall how useful did you find the whole course? *(Please circle one)*

Very useful *Useful* *Not very useful* *Not useful at all*

11. Is there anything else you would like to say?

Thank you.

Please return in the stamped addressed envelope

IWOTCH Participant Feedback Form V1.0 09Oct18. IRAS reference: 199154

This project was funded by the National Institute for Health Research, Health Technology Assessment (project number 14/224/04).
The views and opinions expressed therein are those of the authors and do not necessarily reflect those of the HTA, NIHR, NHS or the Department of Health.

Funded by NIHR

For peer review only

DAY 1 I-WOTCH Fidelity checklists

Group ID: **Date of group session:**

**Day 1: Living with and dealing with pain**	**Date listened**	**Occurred/ Did not**	**Adherence score**	**Competence score**	**Comments**
Session 1 Introduction 15mins					
***Session 2 Pain information approx. 45mins**					
***Session 3 Painkiller information and opioid education approx. 45mins**					
Break					
***Session 4 Acceptance: John's story 20mins**					
Session 5 Attention control and distraction 10mins					
Session 6 Distraction activity – rose drawing 20mins					
Lunch					
***Session 7 Good days, Bad days when is pain bearable and when is it not? 40mins**					
***Session 8 The pain cycle unhelpful emotions and behaviours 40mins**					
Break					
Session 9 Posture					
Session 10 Relaxation and Breathing					
Session 11 Summary of the day					
**Total scores**					

Comments: *e.g. session not recorded so subsequent group same session listened to instead*

For peer review only

I-WOTCH course code: _____ **Reviewer: ___________________________** **Review date: ____________**

Day 1 /Session 2 /Title: Pain Information 30mins

Adherence: of the delivery as per protocol

No.	Item	Adherence	Comments
Intro	Did the facilitator(s) introduce the session?	Yes (2) Partially (1) No (0)	
Step 1	Did the facilitator(s) play the DVD of the biomedical explanation about acute and chronic pain?	Yes (2) Partially (1) No (0)	
	Did the facilitator(s) ask the group Q1 and discuss, "What do you think about this explanation of pain? Is it missing anything?"	Yes (2) Partially (1) No (0)	
Step 2	Did the facilitator(s) present the bio-psycho-social explanation of pain?	Yes (2) Partially (1) No (0)	
	Did the facilitator(s) ask the group Q2 and discuss, "What do you think about this explanation of pain?"	Yes (2) Partially (1) No (0)	
Step 3	Did the facilitator(s) play the DVD of Experiences of living with opioid- treated long term pain?	Yes (2) Partially (1) No (0)	
	Did the facilitator(s) ask the group Q3 and discuss, "What do you think about Caroline's description of living with opioid-treated long-term pain?"	Yes (2) Partially (1) No (0)	
Summary	Did the facilitator(s) consolidate/embed the group's learning at the end of the session? *e.g. reading the summary, putting the session in context*	Yes (2) Partially (1) No (0)	
	Total adherence score (max 16)		
	Percentage adherence score (Total adherence score */16x100)		

Instructions:

When at all possible please rate as 'Yes' or 'No' If 'partially' then write reason in comments box
Questions need not be verbatim (unless specified) as long as content of session is covered.

For peer review only

I-WOTCH course code: _____ **Reviewer:** __________________________ **Review date:** ____________

Comments: For use if sessions; go off track, include items which are not on checklist, contain surprising unforeseen aspects or the facilitation wasn't covered as intended. Also if there was no opportunity to demonstrate the skill listed.

For peer review only

I-WOTCH course code: _____ Reviewer: ___________________________ Review date: ____________

Day 1 /Session 2 /Title: Pain Information and Opioid Education

Competence of the quality of delivery or 'skill' of the facilitators

	Item	Competence measure	Comments (*use box below to expand)*
1	Did the facilitator(s) create opportunities for discussion *e.g. did they; encourage individuals to participate, ask open questions, give enough time for the group to answer (rather than answer their own questions)*	Evident (2)<br>Partially evident (1)<br>Not evident (0)<br>Did not happen in this session (N/A)	
2	Did the facilitator encourage active participation across group members? *e.g. did they encourage quieter members and manage dominant members?*	Evident (2)<br>Partially evident (1)<br>Not evident (0)<br>Did not happen in this session (N/A)	
3	Did the facilitator(s) encourage individual disclosure? *e.g. did they ask different group members to comment or encourage the group to explore issues further (either individually or as a group)?*	Evident (2)<br>Partially evident (1)<br>Not evident (0)<br>Did not happen in this session (N/A)	
4	Did the facilitator(s) validate participants' disclosures? *e.g. Do other people find this/think that? I know how you feel. Sometimes people may feel differently about things.*	Evident (2)<br>Partially evident (1)<br>Not evident (0)<br>Did not happen in this session (N/A)	
5	Did the facilitator(s) give encouraging feedback on participants reported behaviours? *e.g. Did they give appraisal 'that's really good' or 'that's really good but I wonder if…'*	Evident (2)<br>Partially evident (1)<br>Not evident (0)<br>Did not happen in this session (N/A)	
6	Did the facilitator(s) foster a positive group climate? *e.g. did they; use humour, say positive things about people 'that's a helpful comment' 'thank you for sharing that'*	Evident (2)<br>Partially evident (1)<br>Not evident (0)<br>Did not happen in this session (N/A)	
7	Did the facilitator acknowledge and respond appropriately to admissions or statements of low self-efficacy? *e.g. 'yes this can be difficult but…' ideas or examples offered of how this may be done.*	Evident (2)<br>Partially evident (1)<br>Not evident (0)<br>Did not happen in this session (N/A)	
8	Did the facilitator respond appropriately to disclosures of negative events?	Evident (2)<br>Partially evident (1)<br>Not evident (0)<br>Did not happen in this session (N/A)	

For peer review only

I-WOTCH course code: _____ **Reviewer: _________________________** **Review date: ____________**

	Total competence score (max 16)		
	Percentage competence score		

Comments: For use if sessions; go off track, include items which are not on checklist, contain surprising unforeseen aspects or the facilitation wasn't covered as intended. Also if there was no opportunity to demonstrate the skill listed.

For peer review only

BMJ Open

PROCESS EVALUATION PROTOCOL FOR THE IWOTCH STUDY: AN OPIOID TAPERING SUPPORT PROGRAMME FOR PEOPLE WITH CHRONIC NON-MALIGNANT PAIN.

Journal:	*BMJ Open*
Manuscript ID	bmjopen-2019-028998.R1
Article Type:	Protocol
Date Submitted by the Author:	14-Jun-2019
Complete List of Authors:	Nichols, Vivien; University of Warwick, Warwick Clinical Trials Unit<br>Abraham, Charles; University of Melbourne, Faculty of Medicine, Dentistry and Health Sciences<br>Eldabe, Sam; The James Cook University Hospital<br>Sandhu, Harbinder; University of Warwick Warwick Medical School, Clinical Trials Unit<br>Underwood, Martin; Warwick University, Warwick Medical School<br>Seers, Kate; University of Warwick, Warwick Research in Nursing, Warwick Medical School
**Primary Subject Heading**:	General practice / Family practice
Secondary Subject Heading:	Patient-centred medicine
Keywords:	Process Evaluation, Opioid, Protocol, Chronic Non-Malignant pain, Tapering

SCHOLARONE™
Manuscripts

PROCESS EVALUATION PROTOCOL FOR THE IWOTCH STUDY: AN OPIOID TAPERING SUPPORT PROGRAMME FOR PEOPLE WITH CHRONIC NON-MALIGNANT PAIN.

Authors: Vivien Nichols*, Charles Abraham, Sam Eldabe, Harbinder Sandhu, Martin Underwood and Kate Seers on behalf of the IWOTCH team (see acknowledgements)

*Corresponding author

Vivien P Nichols, Warwick Clinical Trials Unit, Division of Health Sciences,

Warwick Medical School, University of Warwick, Coventry CV4 7AL, UK

(+44) 0247657465

V.P.Nichols@warwick.ac.uk

Charles Abraham, School of Psychological Sciences, Faculty of Medicine, Dentistry and Health Sciences,

University of Melbourne, Victoria 3010, Australia

charles.abraham@unimelb.edu.au

Sam Eldabe, Department of Pain Management, The James Cook University Hospital

Marton Road, Middlesbrough TS4 3BW

seldabe@nhs.net

Harbinder Sandhu, Warwick Clinical Trials Unit, Division of Health Sciences,

Warwick Medical School, University of Warwick, Coventry CV4 7AL, UK

Harbinder.K.Sandhu@warwick.ac.uk

Martin Underwood, Warwick Clinical Trials Unit, Division of Health Sciences,

For peer review only - http://bmjopen.bmj.com/site/about/guidelines.xhtml

Warwick Medical School, University of Warwick, Coventry CV4 7AL, UK and University Hospitals Coventry & Warwickshire NHS Trust, University Hospital, Clifford Bridge Road Coventry, CV2 2DX

M.Underwood@warwick.ac.uk

Kate Seers, Warwick Research in Nursing, Warwick Medical School, University of Warwick, Coventry, CV4 7AL, UK.

Kate.Seers@warwick.ac.uk

For peer review only

Word Count: 2,465

Key words: Process Evaluation, Opioids, Protocol, Chronic Non-Malignant Pain, Tapering

For peer review only - http://bmjopen.bmj.com/site/about/guidelines.xhtml

Abstract

Introduction

The Improving the Wellbeing of people with Opioid Treated CHronic pain (I-WOTCH) randomised controlled trial uses a multi component self-management intervention to help people to taper their opioid use. This approach is not widely used and its efficacy is unknown. A process evaluation alongside the trial will help to assess how the intervention was delivered looking at the dose of intervention received and the fidelity of the delivery. We will explore how the intervention may have brought about change through the experiences of the participants receiving and the staff delivering the intervention and whether there were contextual factors involved.

Methods and analysis

A mixed methods process evaluation will assess how the processes of the IWOTCH intervention fared and whether these affected the outcomes. We will collect quantitative data e.g. group attendance analysed with statistical methods. Qualitative data e.g. from interviews and feedback forms will be analysed using framework analysis. We will use a 'following a thread' and a mixed methods matrix for the final integrated analysis.

Ethics and dissemination

The IWOTCH trial and process evaluation have been granted full ethics approval by Yorkshire & The Humber - South Yorkshire Research Ethics Committee on September 13th, 2016 (16/YH/0325). All data has been collected in accordance with data protection guidelines. Participants provide written informed consent for the main trial and all interviewees provide additional written informed consent. The results of the process evaluation will be published and presented at conferences.

For peer review only - http://bmjopen.bmj.com/site/about/guidelines.xhtml

For peer review only

Trial registration:

This trial is registered with an International Standard Randomised Controlled Trial Number (ISRCTN) Register. ISRCTN number: 49470934 (06 Feb 2017).

Article Summary

- Little is known about whether a multi component self-management intervention can help people to taper their opioid use.
- Process evaluation allows for exploration into how a study was implemented, how processes fared and whether these were carried out as intended.
- Qualitative interviews give insight into how people experienced the study both from those delivering and receiving the intervention.
- Using a mixed methods approach will enable us to explore lines of argument across the trial data.

Introduction

The I-WOTCH study

The I-WOTCH study (Improving the Wellbeing of people with Opioid Treated CHronic pain) is a randomised controlled trial (RCT) testing the effectiveness and cost effectiveness of a patient-centred, multicomponent self-management intervention targeting withdrawal of strong opioids among those living with chronic non-malignant pain. Primary outcomes are activities of daily living measured by the Patient-Reported Outcomes Measurement Information System (PROMIS) Pain Interference Short Form (8A) (PROMIS-PI-SF-8A) and opioid dose reduction measured as morphine daily dose equivalent at 12 months follow up. Details of this are included in the I-WOTCH RCT protocol paper.(1) Trial participants are identified from general practice records, using electronic searches and approached by letter. They are randomised into; the control group who receive the 'My Opioid Manager' self-help guide and a relaxation CD or in additionto the intervention group who are invited to attend three days of group activities, two one-

For peer review only - http://bmjopen.bmj.com/site/about/guidelines.xhtml

to-one sessions with a clinical facilitator (usually a nurse) after day two and up to two follow- up telephone calls between day three and the last one-to-one session. I-WOTCH is a multisite trial, requiring standardisation of training and delivery. The intervention is complex and multicomponent, including educational and behaviour change components. Any changes in medication are discussed with participants. All prescriptions continue to be issued by participants' general practitioners. Outcomes will be assessed at four time points, baseline, four, eight and twelve months. The I-WOTCH protocol paper gives greater detail about the study.(1)

Preliminary Work

We did a formative process evaluation as part of an intervention pilot study which found that the randomisation and the control arm seemed acceptable and the paperwork was not reported to be burdensome. There were no reported cases of resentful demoralisation or complaints about the randomisation process.

The delivery of groups and intervention attendance showed that group delivery was feasible, though numbers were lower than expected. Strategies were put in place to improve this in the main study. Once people attended day one attendance was good for the remaining group days and one to one sessions. Those who could not attend the first group session (most often due to work commitments or poor health) were offered a different future group. A member of the process evaluation team (VN) observed one pilot group and reported good group engagement and facilitation of group content. Discussions were well received by the participants attending. Feedback from participants was positive about the course, the most useful aspects being the gaining of new knowledge about opioids and pain within a supportive environment. Participants also said that they found the components of the course which helped them to change their thoughts and attitudes to their pain useful. Things which they would change about the group sessions centred on practical considerations such as the comfort of seating and better sound equipment. This feasibility work helped us to develop our logic model and specific components of the main trial process evaluation such; as our interview topic guides and fidelity paperwork to assess adherence and competence.

For peer review only

For peer review only - http://bmjopen.bmj.com/site/about/guidelines.xhtml

Process evaluation

This paper describes a process evaluation that is being conducted as an integral part of the I-WOTCH trial. We are doing a mixed methods process evaluation based on MRC guidance, to better understand how the intervention works.(2) Key foci of evaluation, as described by Steckler and Linnan are; context, (contextual factors which may affect the implementation), fidelity (whether the intervention was delivered as designed) dose delivered (the amount of the intervention delivered), dose received (the amount of the intervention received by participants) and reach (who the participants are and where they come from).(3) We will assess fidelity, ascertaining whether the trial processes were conducted as per protocol so minimising possible type three errors e.g. when the outcomes of a study do not take into account an inadequate implementation of an intervention.(4) We will identify any delivery which deviates from the original design because these may be important when interpreting the trial results. We will also investigate (i) how the contexts (e.g., different sites) of implementation affected delivery, (ii) how implementation of the intervention was managed and (iii) whether the hypothesised change mechanisms operated as expected. These data can inform replication, development and integration of interventions within routine practice so assisting researchers, commissioners and practitioners.

The process evaluation team (KS, VN and CA) who have expertise in mixed methods approaches to complex health interventions, will work independently of the main trial team during the data collection phase to avoid contamination of trial processes. Findings from the process evaluation may provide insights which could enhance interpretation of the trial results.

Aims:

In summary, the aims of this process evaluation are to investigate:-

1) Experiences of the intervention including enablers of, and barriers to, the intervention facilitating change among participants.
2) Intervention implementation, exploring the dose of the intervention delivered and received and the fidelity of delivery.
3) Change mechanisms assessing whether hypothesised change occurred.

For peer review only - http://bmjopen.bmj.com/site/about/guidelines.xhtml

4) Contextual issues that may affect the outcome or running of the study and/or intervention.

Methods

The aims of the I-WOTCH intervention are to improve participants' quality of life and reduce their use of opioid drugs. We have developed a logic model specific to this intervention guided by Intervention Mapping principles.(5) See figure 1. We have also considered items from a checklist of key features of any group intervention that need to be reported to ensure replication enabling us to identify a series of characteristics to investigate.(6) The intervention includes educational, psychological and behavioural components designed to effect change. Thus the change mechanisms can be conceptualised in terms of the Information, Motivation and Behaviour skills (IMB) model developed by Fisher and Fisher (1992),(7) hypothesising that the intervention will (i) provide useful new information concerning the effects of opioids, (ii) motivate participants to reduce their reliance on opioids and (iii) provide them with new skills to facilitate non-opioid pain control, see figure 2.

We will use a mixed methods approach, using quantitative data collected by the trial team as well as qualitative data collection methods outlined in the sections below which map into our aims.

1) Experiences of the intervention including enablers of, and barriers to, the intervention facilitating change among participants.

Qualitative data will include interviews with participants and those delivering the intervention. Participant feedback forms include qualitative and quantitative data from open and satisfaction questions respectively. See table 1.

Table 1 Interview and participant feedback data

Key Components	Source of Data	Type of Data

For peer review only - http://bmjopen.bmj.com/site/about/guidelines.xhtml

Experiences of participants.<br>Interview topics including:<br>• Responses to receiving the intervention or control<br>• How they felt they were able to use it<br>• How easy or difficult it was to use?<br>• Were some components more challenging than others?<br>• Specific barriers and enablers.<br>• Experience of being in a group. (intervention only)	Participants	Interview recordings and transcripts
Participant feedback forms	Intervention participant forms	Feedback form questions
Experiences of delivering the intervention	Intervention delivery staff (clinical facilitator and lay person with chronic pain or allied health professional)	Interview recordings and transcripts

Interviews

We will interview up to 20 intervention participants and 20 who were allocated to the control arm. These will be purposively sampled to ensure a range of age, gender, location and opioid reduction experience across the two trial arms. They will be interviewed after their final follow up (at 12 months) to minimise possible effects of the interview on the trial findings. The interviews will be semi-structured and held in a convenient local venue to the participant.

Participants will have agreed to be contacted about a possible interview on their initial trial consent form and, after receiving the 12-month follow-up questionnaires, our sample will be sent a patient information leaflet inviting them to take part in a face-to face interview about their experiences of being part of the study. After a week a researcher will contact them by phone to answer any questions and if agreeable book an appointment. A separate informed consent process will be completed at the beginning of each interview.

We will interview up to 20 trained intervention staff across different geographic areas. These include clinical facilitators, (usually a nurse) whose role is to; facilitate groups, see participants for their one-to-one appointments and give them telephone support as required. We will also interview the other group facilitators, either a lay person with experience of opioid use and tapering or an allied health practitioner with an interest in chronic pain conditions. Approach will be by an invitation letter with an information

For peer review only - http://bmjopen.bmj.com/site/about/guidelines.xhtml

leaflet and consent will be taken before the interview. Interviews will be semi structured using a topic guide, and take place after the interventions have been completed.

All interviews will be audio-recorded and transcribed verbatim when all identifiable data will be removed. They will then be checked for accuracy by the interview researcher. Audio recordings will be held in a digitally secure environment with restricted access.

We will analyse the interviews using both thematic analysis and framework analysis.(8, 9) Transcripts will be analyzed using the six steps outlined by Braun and Clarke (2006). After thorough familiarization with the data through listening to all recordings, and reading and re-reading the transcripts, five interviews will be analyzed by coding themes related to the research questions. The emerging lower-level codes will then be grouped into higher-level themes, related to the research questions. All transcripts will then be coded using the hierarchical coding framework, paying attention to any new themes and deviant cases. We will review data related to each code and theme, check and re-coded if necessary, and define themes. Throughout the analysis the analysis team will make reflective analytic memos and hold regular discussion meetings. We will use NVivo qualitative data analysis software; (QSR International Pty Ltd.) to organise the data.

Feedback forms

Feedback forms will be given to intervention participants after their last group or at their second one-to-one session. These forms are anonymous and will be sent back to the Warwick Clinical Trials Unit in a stamped addressed envelope to ensure anonymity. These forms contain quantitative satisfaction questions which will be analysed statistically (see analysis of data section) and open questions which will be analysed using thematic analysis. See appendix 1

2) Intervention implementation exploring dose of the intervention delivered and received, and the fidelity of delivery

We will note the uptake and attendance of the different components of the intervention, to allow assessment of the intervention dose delivered and received, see table 2.

Table 2 Quantitative Data on Dose Delivered and Received

Key Components	Potential Source of Data	Type of Data
Intervention groups	Trial data	Groups run, location and dates

For peer review only - http://bmjopen.bmj.com/site/about/guidelines.xhtml

Numbers attending each component of the three intervention days	Trial data	Attendance sheets per session
Uptake of the one-to-one sessions	Trial data<br>Intervention staff	Intervention trial log<br>Staff interviews
Uptake of the telephone follow up telephone calls.	Trial data<br>Intervention staff	Intervention trial log<br>Staff interviews

Fidelity of Intervention Delivery

Fidelity will be assessed by rating facilitators' adherence to a detailed course manual and competency of delivery as taught in their training. All intervention sessions and one to one consultations will be audio recorded for the purpose of fidelity. This will be carried out by members of the process evaluation team (VN and KS) listening to audio recordings of a sample of group and one-to-one sessions. See table 3

Table 3 Fidelity of Intervention Delivery

Key Components	Source of Data	Type of Data
Assess fidelity of group sessions<br>• Adherence<br>• Competence	Audio recordings of all sessions	Adherence and competence ratings with researcher notes from a selection of sessions
One to one sessions<br>To understand the issues discussed	Audio recordings of all sessions	Adherence and competence ratings with researcher notes from a selection of first and second interviews

The I-WOTCH main study protocol originally had a target of 468 participants (234 of whom will be allocated to the intervention) and anticipated running 24 groups. To ensure a random sample of groups for the fidelity study a statistician using a random number generator will identify; three Day 1 sessions, three Day 2 sessions and three Day 3 sessions from early, middle and late stages of the study. This will ensure we listen to approximately 10% of group sessions across the duration of the study. It was not possible to listen to all the sessions due to pragmatic reasons of time and cost. Through extensive discussions with the team who developed the intervention we decided to pre-specify those sessions which were considered by the team to be key to promoting behaviour change and contain either educational or discussion items. Other sessions which are more practical in nature (e.g. origami for distraction or relaxation) will be difficult to assess from an audio recording as the aim is to promote distraction and discussion or experience a relaxation technique. These will be checked to see if they took place as a minimum requirement of the intervention but will not be rated for facilitator adherence and competence. See table 4.

For peer review only - http://bmjopen.bmj.com/site/about/guidelines.xhtml

For peer review only

Table 4 Course programme with sessions identified for fidelity

**DAY 1**		
Session 1 Introduction		
***Session 2 Pain information**		
***Session 3 Painkiller information and opioid education**		
***Session 4 Acceptance: John's story**		
Session 5 Attention Control and distraction		
Session 6 Distraction activity – rose drawing		
***Session 7 Good days, Bad days when is pain bearable and when is it not?**		
***Session 8 The pain cycle unhelpful emotions and behaviours**		
Session 9 Posture		
Session 10 Relaxation and Breathing		
Session 11 Summary of the day		
**DAY 2**		
Session 12 Reflections from day 1		
***Session 13 Stress-busting – prioritising what's important, action planning, goal setting and pacing**		
***Session 14 Withdrawal symptoms, case studies (Opioid Education 2)**		
Session 15 Distraction activity- origami		
***Session 16 Identifying and overcoming Barriers to change Part 1 – recognising unhelpful thinking**		
***Identifying and overcoming Barriers to change Part 2– reframing negatives to positives**		
Session 17 Mindful attention control		
Session 18 Balance and introduction to stretch		
Session 19 Summary of the Day		
**DAY 3**		
Session 20 Reflections from day 2 and previous week		
***Session 21 Anger, irritability and frustration**		
***Session 22 Relationships Part 1 Getting the most from your healthcare team**		
Session 22 Part 2 Relationships Part 2 Listening skills		
***Session 23 Managing setbacks and non-drug management techniques**		
Session 24 Distraction activity – mindfulness colouring		
Session 25 Stretching muscles that commonly get tight		
Session 26 Mindfulness of thoughts and Senses		
Session 27 Summary of Day 3		
Session 28 Summary of the course		
Legend	***Educational and/or self-management regarding pain or opioid use**	Practical, reflection or summarising sessions
Day 1	**2, 3, 4, 7, 8**	1,5,6,9,10,11
Day 2	**13,14,16**	12,15,17,18,19
Day 3	**21,22 part 1,23,**	20,22 part 2, 24,25,26,27,28

To assess the fidelity of the intervention we will assess two aspects of intervention delivery; adherence to the intervention manual and competency of the facilitators. A member of the process evaluation team (VN) will listen to the relevant recordings and score adherence and competence using a specially devised checklist based upon components specified in the manual items and training protocol for intervention facilitators. See appendices 2 and 3 for examples.

We will also rate one of the first or second one-to-one nurse consultations per group N=24. We will double rate 10% of these sampled group and one-to-one sessions by a second member of the team (KS) to assess

For peer review only - http://bmjopen.bmj.com/site/about/guidelines.xhtml

inter-rater reliability and to ensure rigour. Percentage scores will be given for adherence and competence per session and the findings analysed using standard statistical methods.(see mixed methods analysis)

3) Change Mechanisms assessing whether hypothesised change occurred.

We will administer self-report questions within the IWOTCH RCT questionnaires (at baseline, four, eight, and twelve months) to track possible change mechanisms. Specifically we will assess participants' (i) motivation to reduce opioid use before and after the intervention, (ii) expectations of success in opioid reduction and (iii) confidence (or self-efficacy (10)) in relation to opioid reduction prior to receiving the intervention and (iv) perceived intervention efficacy before and after participation, see table 5. These data will be analysed using standard statistical methods, including t tests and ANCOVA.

Table 5 Motivation Expectations, Self-Efficacy and Perceived Intervention Efficacy Questions

Baseline Motivation (baseline and follow up)
I want to reduce my opioid use
(Not at all, by a little, by half, so I only use a little, so I use no opioids)

Baseline Expectation (baseline only)
I expect that, in 4 months' time, I will have reduced my opioid use:
(Not at all, by a little, by half, so I only use a little, so I use no opioids)

Baseline Self-Efficacy (baseline only)
I am confident I could reduce my opioid use a lot over 4 months
(Not at all confident, somewhat confident, fairly confident, strongly confident, completely confident)

Perceived Intervention Efficacy (baseline and follow up)
Baseline
I feel that involvement in this study can help me to reduce my opioid use
Not at all, by a little, by half, so I only use a little, so I use no opioids

Follow up
I feel that involvement in this study has helped me to reduce my opioid use
Not at all, by a little, by half, so I only use a little, so I use no opioids

For peer review only - http://bmjopen.bmj.com/site/about/guidelines.xhtml

4) Contextual issues that may affect the outcome or running of the study and/or intervention

Contextual factors may be found in the data collected above may influence change and outcomes. We will explore this as the need arises from the data as it may be a ‘thread to follow’ (see paragraph below) or an integral part of a section of the analysis.

Mixed Methods Analysis

Quantitative data will be analysed statistically to produce appropriate descriptive statistics, tables, charts or figures. Data from quantitative and qualitative findings will be integrated as outlined by O’Cathain et al.(11) We will use both ‘following a thread’ which involves selecting a question or component from one aspect of the findings and following across, and ‘mixed methods matrix’ where, for example, responses on quantitative scales can be compared to interview transcript, and data on each case can be concisely stated and recorded on a matrix. For detailed explanation of ‘following a thread’, we refer the reader to O’Cathain.(11)

Patient and Public Involvement

This process evaluation is part of the I-WOTCH study which has patient and public involvement with regard to input into its design as well as the ongoing running of the study which is described more fully elsewhere.(1) Patient participant interviews are an integral part of this process evaluation. All trial participants will be notified of the study findings via a study newsletter and a lay summary will be available on the study website.

Trial Status

The I-WOTCH study began recruitment in May 2017 and anticipate groups will be running into February 2019, data collection will be completed around February 2020 and they expect the final report for the funders will be submitted mid-2020.

For peer review only - http://bmjopen.bmj.com/site/about/guidelines.xhtml

Ethics and Dissemination

We intend to publish the process evaluation findings in peer reviewed journals and details of the main trial ethics and dissemination are outlined in the main trial protocol.(1)

The IWOTCH trial and process evaluation have been granted full ethics approval by Yorkshire & The Humber - South Yorkshire Research Ethics Committee on September 13th, 2016 (16/YH/0325).

References

1. Sandhu H, Abraham C, Alleyne S, et al. Testing a support programme for opioid tapering for people with chronic non-malignant pain: The I-WOTCH Study protocol submitted to BMJopen2018.
2. Moore GF, Audrey S, Barker M, et al. Process evaluation of complex interventions: Medical Research Council guidance. BMJ2015;350:h1258.
3. Steckler A, Linnan L. Process Evaluation for Public health Interventions and research. San Fransisco: Jossey-Bass; 2002.
4. Basch CE, Sliepcevich EM, Gold RS, et al. Avoiding type III errors in health education program evaluations: a case study. Health Educ Q1985;12(4):315-31.
5. Bartholomew Eldredge LK, Markham CM, Ruiter RAC, et al. Planning health promotion programs: Jossey-Bass Inc.; 2016 2016.
6. Borek AJ, Abraham C, Smith JR, et al. A checklist to improve reporting of group-based behaviour-change interventions. BMC Public Health2015;15:963.
7. Fisher JDF, W.A. The Information-Motivation-Behavioural Skills Model. In: DiClemente R, Crosby R, Kegler M, editors. Emerging theories in health promotion practice and research: Strategies for improving public health. San Fransisco: Jossey-Bass; 2002. p. 40-7.
8. Ritchie J, Spencer L. Qualitative data analysis for applied policy research. The qualitative researcher's companion2002;573(2002):305-29.
9. Braun V, Clarke V. Using thematic analysis in psychology. Qualitative Research in Psychology2006;3(2):77-101.
10. Bandura A. Self-efficacy: toward a unifying theory of behavioral change. Psychol Rev1977;84(2):191-215.
11. O'Cathain A, Murphy E, Nicholl J. Three techniques for integrating data in mixed methods studies. BMJ (Online)2010;341(7783):1147-50.

Authors' contributions

KS, MU, SE and HS conceived the original study design. VN, KS and CA have developed the study design, and plan for data collection and analysis. All authors have provided critical revisions to the manuscript and approved the final manuscript.

For peer review only - http://bmjopen.bmj.com/site/about/guidelines.xhtml

Funding

This project is funded by the National Institute for Health Research (NIHR), Health Technology Assessment (HTA) (project number 14/224/04). The views and opinions expressed therein are those of the authors and do not necessarily reflect those of the HTA, NIHR, NHS or the Department of Health.

Competing interests statement.

SE is investigator on a number of NIHR and industry sponsored studies. He received travel expenses for speaking at conferences from the professional organisations. SE consults for Medtronic, Abbott, Boston Scientific and Mainstay Medical, none in relation to opioids. SE is chair of the BPS Science and Research Committee. SE is deputy Chair of the NIHR CRN Anaesthesia Pain and Perioperative Medicine National Specialty Group. SE's department has received fellowship funding from Medtronic as well as nurse funding from Abbott.

HS is director of Health Psychology Services Ltd, providing psychological services for a range of health related conditions.

MU was Chair of the NICE accreditation advisory committee until March 2017 for which he received a fee. He is chief investigator or co-investigator on multiple previous and current research grants from the UK National Institute for Health Research, Arthritis Research UK and is a co-investigator on grants funded by the Australian NHMRC. He is an NIHR Senior Investigator. He has received travel expenses for speaking at conferences from the professional organisations hosting the conferences. He is a director and shareholder of Clinvivo Ltd that provides electronic data collection for health services research. He is part of an academic partnership with Serco Ltd related to return to work initiatives. He is a co-investigator on a study receiving support in kind from Orthospace Ltd. He is an editor of the NIHR journal series, and a member of the NIHR Journal Editors Group, for which he receives a fee.

KS received grant funding as PI and CoI from NIHR for other projects and was on the NIHR HS&DR Funding Board until January 2018.

For peer review only

Acknowledgements

This manuscript was written on behalf of the I-WOTCH team:

Sharisse Alleyne, University of Warwick
Shyam Balasubramanian, Department of Anaesthesia & Pain Medicine University Hospitals Coventry and Warwickshire NHS Trust
Lauren Betteley, University of Warwick
Katie Booth, University of Warwick
Dawn Carnes, Barts & The London Queen Mary's School of Medicine & Dentistry
Andrea Dompieri Furlan, Toronto Rehabilitation Institute, University Health Network, Canada
Kirstie Haywood, University of Warwick
Ranjit Lall, University of Warwick
Andrea Manca, The University of York
Dipesh Mistry, University of Warwick
Jennifer Noyes, The James Cook University Hospital
Anisur Rahman, University College London
Jane Shaw, The James Cook University Hospital
Nicole Kit Yee Tang, University of Warwick
Stephanie Taylor, Barts & The London Queen Mary's School of Medicine & Dentistry
Colin Tysall, University/User Teaching and Research Action Partnership, University of Warwick
Emma Withers, University of Warwick
Martin Underwood, University of Warwick
Cynthia Paola Iglesis Urrutia, The University of York

Figure 1 Logic Model

Figure 2 Information Motivation Behavioural skills model

For peer review only

Figure 1 Logic model

The problem	Intervention Aims	Intervention	Theory and Guidance	Interim Targets	Desired Outcomes
People with chronic non-malignant pain are taking opioids, which have side effects and are not effective in the long term.	To test the effectiveness and cost effectiveness of a patient-centred multicomponent self-management intervention targeting withdrawal of strong opioids on activities of daily living for people living with chronic non-malignant pain	**Manualised Intervention Delivery**<br>**Core pain management topics:**<br>• Acute versus Chronic pain<br>• Acceptance<br>• Attention Control and distraction<br>• the pain cycle<br>• Posture and movement advice<br>• Relaxation techniques<br>• Stress busting for health action planning, problem solving, pacing, SMART goal setting<br>• identifying and overcoming barriers to change<br>• Mindfulness<br>• Anger, irritability and frustration<br>• Communication Skills<br><br>**Core opioid specific topics:**<br>• The rationale of prescribing in chronic pain<br>• Opioid induced tolerance and need for dose escalation<br>• Evidence of usefulness of opioids short and long term<br>• Side effects of opioids short term and long term<br>• Case studies of successful discontinued opioid therapy<br>• Opioid withdrawal symptoms<br>• Advantages of slow supervised tapering<br>• Symptom management during tapering<br>• Pain control after opioids	Theory of Planned Behaviour<br><br>Social Cognitive Theory<br><br>Information Motivation and Behavioural (IMB model) skills<br><br>Patient Centred Communication<br><br>Motivational Interviewing	**Staff Training**<br>To facilitate groups, deliver individual tapering consultations and telephone support in an inclusive and non-judgemental manner<br>**Individual participant changes:**<br>a Knowledge of: opioids, withdrawal effects, chronic pain<br>b Fostering change: self-validation, legitimising pain, normalising expectations<br>c Motivation to change by: Improved self-efficacy, effective tapering<br>d Skills:<br>• General Self-Regulation<br>*Psychological skills*<br>*Identify reasons for negative emotions (anger /frustration /irritable)*<br>*Identify problems and solutions, barriers to change*<br>*Recognise errors in thinking/automatic thoughts*<br>*Goal setting, goal review*<br>*Physical skills*<br>*Promote body awareness, posture*<br>*Reduce muscle tension*<br>*Body awareness and core strength*<br>*Relaxation-contract relax*<br>• Pain Self-Regulation<br>Understand that pain and mood are linked – when is pain bearable and when not bearable.<br>Understanding of pain cycle, unhelpful emotions and behaviours<br>Using mind to relieve pain does not mean pain in mind<br>Distraction whilst relaxed<br>Focus mind away from pain<br>Mindfulness for pain<br>Managing flare ups<br>Need for stretching<br>• Communication Skills<br>How to communicate with General Practitioners (GPs) and Health Care Professionals (HCPs)<br>Listening skills - Active and giving feedback in communication-reward for help.	**Primary outcomes:**<br>Patient-Reported Outcomes Measurement Information System (PROMIS) Pain Interference Short Form (8A)(PROMIS-PI-SF-8A)<br><br>Daily morphine equivalent opioid dose

For peer review only

Figure 2. The Information-Motivation-Behavioural Skills Model

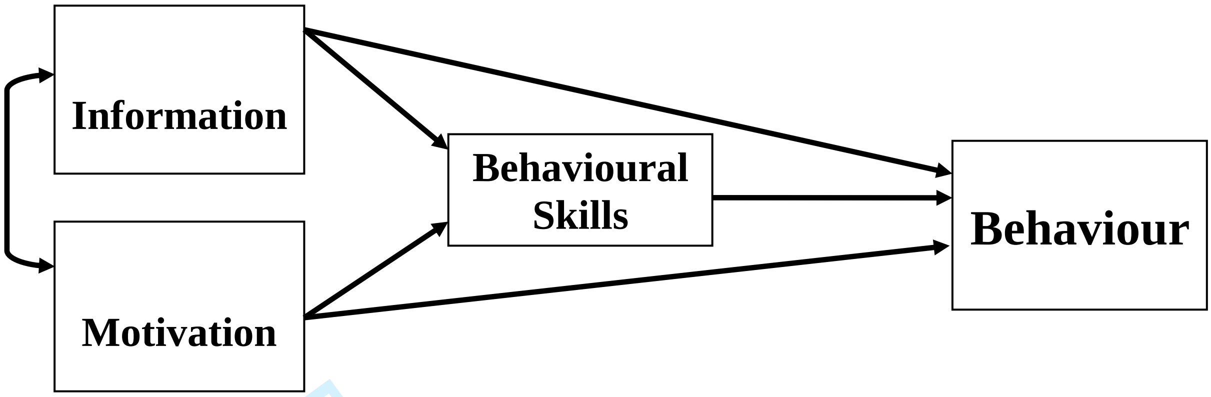

From J. D. Fisher and W. A. Fisher (1992). Changing AIDS risk behavior. Psychological Bulletin, 111, 455–74. Copyright by APA. Reprinted with permission.

For peer review only

Group venue: ________________________________

Date: ________________________________

I-WOTCH Feedback form

Thank you for attending the I-WOTCH course. Please complete the feedback and short questionnaire below. This information will be used to evaluate the support programme. Please note that these forms are anonymous and will be sent back to the study team.

1. Were the aims of the course made clear? Please circle one

Yes *No* *Don't know*

2. What were the three most useful things on this course?

1)..

2)..

3)..

3. What three things would you suggest to make this course better for future participants?

1) ....

2) ....

3) ....

4. How confident do you feel that the course content will help you personally? *(Please circle one)*

Very confident *Confident* *Not very confident* *Not confident at all*

5. How confident do you feel that you will be able to use this in the future? *(Please circle one)*

Very confident *Confident* *Not very confident* *Not confident at all*

PLEASE TURN OVER THE PAGE FOR REMAINING QUESTIONS

IWOTCH Participant Feedback Form V1.0 09Oct18. IRAS reference: 199154

This project was funded by the National Institute for Health Research, Health Technology Assessment (project number 14/224/04).
The views and opinions expressed therein are those of the authors and do not necessarily reflect those of the HTA, NIHR, NHS or the Department of Health.

Funded by NIHR

For peer review only

I-WATCH
IMPROVING THE WELLBEING OF PEOPLE WITH OPIOID TREATED CHRONIC PAIN

Group venue: ______________________________

Date: ______________________________

6. Overall were the facilitators: *(Please circle one)*

Very good *Good* *Satisfactory* *Poor*

7. Overall were the handouts: *(Please circle one)*

Very good *Good* *Satisfactory* *Poor*

8. How did you find the face to face meeting with the nurse? *(Please circle one)*

Very useful *Useful* *Not very useful* *Not useful at all*

9. How did you find the telephone calls with the nurse? *(Please circle one)*

Very useful *Useful* *Not very useful* *Not useful at all*

10. Overall how useful did you find the whole course? *(Please circle one)*

Very useful *Useful* *Not very useful* *Not useful at all*

11. Is there anything else you would like to say?

Thank you.

Please return in the stamped addressed envelope

IWOTCH Participant Feedback Form V1.0 09Oct18. IRAS reference: 199154

This project was funded by the National Institute for Health Research, Health Technology Assessment (project number 14/224/04).
The views and opinions expressed therein are those of the authors and do not necessarily reflect those of the HTA, NIHR, NHS or the Department of Health.

Funded by NIHR

For peer review only

DAY 1 I-WOTCH Fidelity checklists

Group ID: **Date of group session:**

**Day 1: Living with and dealing with pain**	**Date listened**	**Occurred/ Did not**	**Adherence score**	**Competence score**	**Comments**
Session 1 Introduction 15mins					
***Session 2 Pain information approx. 45mins**					
***Session 3 Painkiller information and opioid education approx. 45mins**					
Break					
***Session 4 Acceptance: John's story 20mins**					
Session 5 Attention control and distraction 10mins					
Session 6 Distraction activity – rose drawing 20mins					
Lunch					
***Session 7 Good days, Bad days when is pain bearable and when is it not? 40mins**					
***Session 8 The pain cycle unhelpful emotions and behaviours 40mins**					
Break					
Session 9 Posture					
Session 10 Relaxation and Breathing					
Session 11 Summary of the day					
**Total scores**					

Comments: *e.g. session not recorded so subsequent group same session listened to instead*

I-WOTCH course code: _____ Reviewer: __________________________ Review date: ____________

Day 1 /Session 2 /Title: Pain Information 30mins

Adherence: of the delivery as per protocol

No.	Item	Adherence	Comments
Intro	Did the facilitator(s) introduce the session?	Yes (2) Partially (1) No (0)	
Step 1	Did the facilitator(s) play the DVD of the biomedical explanation about acute and chronic pain?	Yes (2) Partially (1) No (0)	
	Did the facilitator(s) ask the group Q1 and discuss, "What do you think about this explanation of pain? Is it missing anything?"	Yes (2) Partially (1) No (0)	
Step 2	Did the facilitator(s) present the bio-psycho-social explanation of pain?	Yes (2) Partially (1) No (0)	
	Did the facilitator(s) ask the group Q2 and discuss, "What do you think about this explanation of pain?"	Yes (2) Partially (1) No (0)	
Step 3	Did the facilitator(s) play the DVD of Experiences of living with opioid- treated long term pain?	Yes (2) Partially (1) No (0)	
	Did the facilitator(s) ask the group Q3 and discuss, "What do you think about Caroline's description of living with opioid-treated long-term pain?"	Yes (2) Partially (1) No (0)	
Summary	Did the facilitator(s) consolidate/embed the group's learning at the end of the session? *e.g. reading the summary, putting the session in context*	Yes (2) Partially (1) No (0)	
	Total adherence score (max 16)		
	Percentage adherence score (Total adherence score */16x100)		

Instructions:

When at all possible please rate as 'Yes' or 'No' If 'partially' then write reason in comments box
Questions need not be verbatim (unless specified) as long as content of session is covered.

For peer review only

I-WOTCH course code: _____ **Reviewer: _________________________** **Review date: ____________**

Comments: For use if sessions; go off track, include items which are not on checklist, contain surprising unforeseen aspects or the facilitation wasn't covered as intended. Also if there was no opportunity to demonstrate the skill listed.

For peer review only

I-WOTCH course code: _____ **Reviewer: __________________________** **Review date: ____________**

Day 1 /Session 2 /Title: Pain Information and Opioid Education

Competence **of the quality of delivery or 'skill' of the facilitators**

	Item	Competence measure	Comments (*use box below to expand*)
1	Did the facilitator(s) create opportunities for discussion *e.g. did they; encourage individuals to participate, ask open questions, give enough time for the group to answer (rather than answer their own questions)*	Evident (2)<br>Partially evident (1)<br>Not evident (0)<br>Did not happen in this session (N/A)	
2	Did the facilitator encourage active participation across group members? *e.g. did they encourage quieter members and manage dominant members?*	Evident (2)<br>Partially evident (1)<br>Not evident (0)<br>Did not happen in this session (N/A)	
3	Did the facilitator(s) encourage individual disclosure? *e.g. did they ask different group members to comment or encourage the group to explore issues further (either individually or as a group)?*	Evident (2)<br>Partially evident (1)<br>Not evident (0)<br>Did not happen in this session (N/A)	
4	Did the facilitator(s) validate participants' disclosures? *e.g. Do other people find this/think that? I know how you feel. Sometimes people may feel differently about things.*	Evident (2)<br>Partially evident (1)<br>Not evident (0)<br>Did not happen in this session (N/A)	
5	Did the facilitator(s) give encouraging feedback on participants reported behaviours? *e.g. Did they give appraisal 'that's really good' or 'that's really good but I wonder if…'*	Evident (2)<br>Partially evident (1)<br>Not evident (0)<br>Did not happen in this session (N/A)	
6	Did the facilitator(s) foster a positive group climate? *e.g. did they; use humour, say positive things about people 'that's a helpful comment' 'thank you for sharing that'*	Evident (2)<br>Partially evident (1)<br>Not evident (0)<br>Did not happen in this session (N/A)	
7	Did the facilitator acknowledge and respond appropriately to admissions or statements of low self-efficacy? *e.g. 'yes this can be difficult but…' ideas or examples offered of how this may be done.*	Evident (2)<br>Partially evident (1)<br>Not evident (0)<br>Did not happen in this session (N/A)	
8	Did the facilitator respond appropriately to disclosures of negative events?	Evident (2)<br>Partially evident (1)<br>Not evident (0)<br>Did not happen in this session (N/A)	

For peer review only

I-WOTCH course code: _____ **Reviewer: _________________________** **Review date: ____________**

	Total competence score (max 16)		
	Percentage competence score		

Comments: For use if sessions; go off track, include items which are not on checklist, contain surprising unforeseen aspects or the facilitation wasn't covered as intended. Also if there was no opportunity to demonstrate the skill listed.

For peer review only

BMJ Open

PROCESS EVALUATION PROTOCOL FOR THE I-WOTCH STUDY: AN OPIOID TAPERING SUPPORT PROGRAMME FOR PEOPLE WITH CHRONIC NON-MALIGNANT PAIN.

Journal:	*BMJ Open*
Manuscript ID	bmjopen-2019-028998.R2
Article Type:	Protocol
Date Submitted by the Author:	20-Aug-2019

Complete List of Authors:	Nichols, Vivien; University of Warwick, Warwick Clinical Trials Unit, Warwick Medical School<br>Abraham, Charles; University of Melbourne, Faculty of Medicine, Dentistry and Health Sciences<br>Eldabe, Sam; The James Cook University Hospital, Department of Pain Medicine,<br>Sandhu, Harbinder; University of Warwick, Warwick Clinical Trials Unit, Warwick Medical School<br>Underwood, Martin; University of Warwick, Warwick Clinical Trials Unit, Warwick Medical School<br>Seers, Kate; University of Warwick, Warwick Research in Nursing, Division of Health Sciences, Warwick Medical School

**Primary Subject Heading**:	General practice / Family practice
Secondary Subject Heading:	Patient-centred medicine
Keywords:	Process Evaluation, Opioid, Protocol, Chronic Non-Malignant pain, Tapering

SCHOLARONE™ Manuscripts

PROCESS EVALUATION PROTOCOL FOR THE I-WOTCH STUDY: AN OPIOID TAPERING SUPPORT PROGRAMME FOR PEOPLE WITH CHRONIC NON-MALIGNANT PAIN.

Authors: Vivien Nichols*, Charles Abraham, Sam Eldabe, Harbinder Sandhu, Martin Underwood and Kate Seers on behalf of the I-WOTCH team (see acknowledgements)

*Corresponding author

Vivien P Nichols, Warwick Clinical Trials Unit, Warwick Medical School,

University of Warwick, Coventry CV4 7AL, UK

(+44) 0247657465

V.P.Nichols@warwick.ac.uk

Charles Abraham, School of Psychological Sciences, Faculty of Medicine, Dentistry and Health Sciences,

University of Melbourne, Victoria 3010, Australia

charles.abraham@unimelb.edu.au

Sam Eldabe, Department of Pain Medicine, The James Cook University Hospital

Marton Road, Middlesbrough TS4 3BW

seldabe@nhs.net

Harbinder Sandhu, Warwick Clinical Trials Unit, Warwick Medical School, University of Warwick,

Coventry CV4 7AL, UK

Harbinder.K.Sandhu@warwick.ac.uk

For peer review only

For peer review only - http://bmjopen.bmj.com/site/about/guidelines.xhtml

Martin Underwood, Warwick Clinical Trials Unit, Warwick Medical School, University of Warwick, Coventry CV4 7AL, UK

M.Underwood@warwick.ac.uk

Kate Seers, Warwick Research in Nursing, Division of Health Sciences, Warwick Medical School, University of Warwick, Coventry, CV4 7AL, UK.

Kate.Seers@warwick.ac.uk

Word Count: 2,494

Key words: Process Evaluation, Opioids, Protocol, Chronic Non-Malignant Pain, Tapering

For peer review only

Abstract

Introduction

The Improving the Wellbeing of people with Opioid Treated CHronic pain (I-WOTCH) randomised controlled trial uses a multi component self-management intervention to help people to taper their opioid use. This approach is not widely used and its efficacy is unknown. A process evaluation alongside the trial will help to assess how the intervention was delivered looking at the dose of intervention received and the fidelity of the delivery. We will explore how the intervention may have brought about change through the experiences of the participants receiving and the staff delivering the intervention and whether there were contextual factors involved.

Methods and analysis

A mixed methods process evaluation will assess how the processes of the I-WOTCH intervention fared and whether these affected the outcomes. We will collect quantitative data e.g. group attendance analysed with statistical methods. Qualitative data e.g. from interviews and feedback forms will be analysed using framework analysis. We will use a 'following a thread' and a mixed methods matrix for the final integrated analysis.

Ethics and dissemination

The I-WOTCH trial and process evaluation have been granted full ethics approval by Yorkshire & The Humber - South Yorkshire Research Ethics Committee on September 13th, 2016 (16/YH/0325). All data has been collected in accordance with data protection guidelines. Participants provide written informed consent for the main trial and all interviewees provide additional written informed consent. The results of the process evaluation will be published and presented at conferences.

For peer review only - http://bmjopen.bmj.com/site/about/guidelines.xhtml

Trial registration:

This trial is registered with an International Standard Randomised Controlled Trial Number (ISRCTN) Register. ISRCTN number: 49470934 (06 Feb 2017).

Article Summary

- Little is known about whether a multi component self-management intervention can help people to taper their opioid use.
- Process evaluation allows for exploration into how a study was implemented, how processes fared and whether these were carried out as intended.
- Qualitative interviews give insight into how people experienced the study both from those delivering and receiving the intervention.
- Using a mixed methods approach will enable us to explore lines of argument across the trial data.

Introduction

The I-WOTCH study

The I-WOTCH study (Improving the Wellbeing of people with Opioid Treated CHronic pain) is a randomised controlled trial (RCT) testing the effectiveness and cost effectiveness of a patient-centred, multicomponent self-management intervention targeting withdrawal of strong opioids among those living with chronic non-malignant pain. Primary outcomes are activities of daily living measured by the Patient-Reported Outcomes Measurement Information System (PROMIS) Pain Interference Short Form (8A) (PROMIS-PI-SF-8A) and opioid dose reduction measured as morphine daily dose equivalent at 12 months follow up. Details of this are included in the I-WOTCH RCT protocol paper.(1) Trial participants are identified from general practice records, using electronic searches and approached by letter. They are randomised into; the control group who receive the 'My Opioid Manager' self-help guide and a relaxation CD or in addition to the intervention group who are invited to attend three days of group activities, two one-

For peer review only - http://bmjopen.bmj.com/site/about/guidelines.xhtml

to-one sessions with a clinical facilitator (usually a nurse) after day two and up to two follow- up telephone calls between day three and the last one-to-one session. I-WOTCH is a multisite trial, requiring standardisation of training and delivery. The intervention is complex and multicomponent, including educational and behaviour change components. Any changes in medication are discussed with participants. All prescriptions continue to be issued by participants' general practitioners. Outcomes will be assessed at four time points, baseline, four, eight and twelve months. The I-WOTCH protocol paper gives greater detail about the study.(1)

Preliminary Work

We did a formative process evaluation as part of an intervention pilot study which found that the randomisation and the control arm seemed acceptable and the paperwork was not reported to be burdensome. There were no reported cases of resentful demoralisation or complaints about the randomisation process.

The delivery of groups and intervention attendance showed that group delivery was feasible, though numbers were lower than expected. Strategies were put in place to improve this in the main study. Once people attended day one, attendance was good for the remaining group days and one to one sessions. Those who could not attend the first group session (most often due to work commitments or poor health) were offered a different future group. A member of the process evaluation team (VN) observed one pilot group and reported good group engagement and facilitation of group content. Discussions were well received by the participants attending. Feedback from participants was positive about the course, the most useful aspects being the gaining of new knowledge about opioids and pain within a supportive environment. Participants also said that they found the components of the course which helped them to change their thoughts and attitudes to their pain useful. Things which they would change about the group sessions centred on practical considerations such as the comfort of seating and better sound equipment. This feasibility work helped us to develop our logic model and specific components of the main trial process evaluation such; as our interview topic guides and fidelity paperwork to assess adherence and competence.

For peer review only - http://bmjopen.bmj.com/site/about/guidelines.xhtml

Process evaluation

This paper describes a process evaluation that is being conducted as an integral part of the I-WOTCH trial. We are doing a mixed methods process evaluation based on MRC guidance, to better understand how the intervention works.(2) Key foci of evaluation, as described by Steckler and Linnan are; context, (contextual factors which may affect the implementation), fidelity (whether the intervention was delivered as designed) dose delivered (the amount of the intervention delivered), dose received (the amount of the intervention received by participants) and reach (who the participants are and where they come from).(3) We will assess fidelity, ascertaining whether the trial processes were conducted as per protocol so minimising possible type three errors e.g. when the outcomes of a study do not take into account an inadequate implementation of an intervention.(4) We will identify any delivery which deviates from the original design because these may be important when interpreting the trial results. We will also investigate (i) how the contexts (e.g., different sites) of implementation affected delivery, (ii) how implementation of the intervention was managed and (iii) whether the hypothesised change mechanisms operated as expected. These data can inform replication, development and integration of interventions within routine practice so assisting researchers, commissioners and practitioners.

The process evaluation team (KS, VN and CA) who have expertise in mixed methods approaches to complex health interventions, will work independently of the main trial team during the data collection phase to avoid contamination of trial processes. Findings from the process evaluation may provide insights which could enhance interpretation of the trial results.

Aims:

In summary, the aims of this process evaluation are to investigate:-

1) Experiences of the intervention including enablers of, and barriers to, the intervention facilitating change among participants.
2) Intervention implementation, exploring the dose of the intervention delivered and received and the fidelity of delivery.
3) Change mechanisms assessing whether hypothesised change occurred.

For peer review only - http://bmjopen.bmj.com/site/about/guidelines.xhtml

4) Contextual issues that may affect the outcome or running of the study and/or intervention.

Methods

The aims of the I-WOTCH intervention are to improve participants' quality of life and reduce their use of opioid drugs. We have developed a logic model specific to this intervention guided by Intervention Mapping principles.(5) See figure 1. We have also considered items from a checklist of key features of any group intervention that need to be reported to ensure replication enabling us to identify a series of characteristics to investigate.(6) The intervention includes educational, psychological and behavioural components designed to effect change. Thus the change mechanisms can be conceptualised in terms of the Information, Motivation and Behaviour skills (IMB) model developed by Fisher and Fisher (1992),(7) hypothesising that the intervention will (i) provide useful new information concerning the effects of opioids, (ii) motivate participants to reduce their reliance on opioids and (iii) provide them with new skills to facilitate non-opioid pain control, see figure 2.

We will use a mixed methods approach, using quantitative data collected by the trial team as well as qualitative data collection methods outlined in the sections below which map into our aims.

1) Experiences of the intervention including enablers of, and barriers to, the intervention facilitating change among participants.

Qualitative data will include interviews with participants and those delivering the intervention. Participant feedback forms include qualitative and quantitative data from open and satisfaction questions respectively. See table 1.

Table 1 Interview and participant feedback data

Key Components	Source of Data	Type of Data

For peer review only - http://bmjopen.bmj.com/site/about/guidelines.xhtml

Experiences of participants.<br>Interview topics including:<br>• Responses to receiving the intervention or control<br>• How they felt they were able to use it<br>• How easy or difficult it was to use?<br>• Were some components more challenging than others?<br>• Specific barriers and enablers.<br>• Experience of being in a group. (intervention only)	Participants	Interview recordings and transcripts
Participant feedback forms	Intervention participant forms	Feedback form questions
Experiences of delivering the intervention	Intervention delivery staff<br>(clinical facilitator and lay person with chronic pain or allied health professional)	Interview recordings and transcripts

Interviews

We will interview up to 20 intervention participants and 20 who were allocated to the control arm. These will be purposively sampled to ensure a range of age, gender, location and opioid reduction experience across the two trial arms. They will be interviewed after their final follow up (at 12 months) to minimise possible effects of the interview on the trial findings. The interviews will be semi-structured and held in a convenient local venue to the participant.

Participants will have agreed to be contacted about a possible interview on their initial trial consent form and, after receiving the 12-month follow-up questionnaires, our sample will be sent a patient information leaflet inviting them to take part in a face-to face interview about their experiences of being part of the study. After a week a researcher will contact them by phone to answer any questions and if agreeable book an appointment. A separate informed consent process will be completed at the beginning of each interview.

We will interview up to 20 trained intervention staff across different geographic areas. These include clinical facilitators, (usually a nurse) whose role is to; facilitate groups, see participants for their one-to-one appointments and give them telephone support as required. We will also interview the other group facilitators, either a lay person with experience of opioid use and tapering or an allied health practitioner with an interest in chronic pain conditions. Approach will be by an invitation letter with an information

For peer review only - http://bmjopen.bmj.com/site/about/guidelines.xhtml

leaflet and consent will be taken before the interview. Interviews will be semi structured using a topic guide, and take place after the interventions have been completed.

All interviews will be audio-recorded and transcribed verbatim when all identifiable data will be removed. They will then be checked for accuracy by the interview researcher. Audio recordings will be held in a digitally secure environment with restricted access.

We will analyse the interviews using both thematic analysis and framework analysis.(8, 9) Transcripts will be analyzed using the six steps outlined by Braun and Clarke (2006). After thorough familiarization with the data through listening to all recordings, and reading and re-reading the transcripts, five interviews will be analyzed by coding themes related to the research questions. The emerging lower-level codes will then be grouped into higher-level themes, related to the research questions. All transcripts will then be coded using the hierarchical coding framework, paying attention to any new themes and deviant cases. We will review data related to each code and theme, check and re-coded if necessary, and define themes. Throughout the analysis the analysis team will make reflective analytic memos and hold regular discussion meetings. We will use NVivo qualitative data analysis software; (QSR International Pty Ltd.) to organise the data.

Feedback forms

Feedback forms will be given to intervention participants after their last group or at their second one-to-one session. These forms are anonymous and will be sent back to the Warwick Clinical Trials Unit in a stamped addressed envelope to ensure anonymity. These forms contain quantitative satisfaction questions which will be analysed statistically (see analysis of data section) and open questions which will be analysed using thematic analysis. See appendix 1

2) Intervention implementation exploring dose of the intervention delivered and received, and the fidelity of delivery

We will note the uptake and attendance of the different components of the intervention, to allow assessment of the intervention dose delivered and received, see table 2.

Table 2 Quantitative Data on Dose Delivered and Received

Key Components	Potential Source of Data	Type of Data
Intervention groups	Trial data	Groups run, location and dates

For peer review only - http://bmjopen.bmj.com/site/about/guidelines.xhtml

Numbers attending each component of the three intervention days	Trial data	Attendance sheets per session
Uptake of the one-to-one sessions	Trial data<br>Intervention staff	Intervention trial log<br>Staff interviews
Uptake of the telephone follow up telephone calls.	Trial data<br>Intervention staff	Intervention trial log<br>Staff interviews

Fidelity of Intervention Delivery

Fidelity will be assessed by rating facilitators' adherence to a detailed course manual and competency of delivery as taught in their training. All intervention sessions and one to one consultations will be audio recorded for the purpose of fidelity. This will be carried out by members of the process evaluation team (VN and KS) listening to audio recordings of a sample of group and one-to-one sessions. See table 3

Table 3 Fidelity of Intervention Delivery

Key Components	Source of Data	Type of Data
Assess fidelity of group sessions<br>• Adherence<br>• Competence	Audio recordings of all sessions	Adherence and competence ratings with researcher notes from a selection of sessions
One to one sessions<br>To understand the issues discussed	Audio recordings of all sessions	Adherence and competence ratings with researcher notes from a selection of first and second interviews

The I-WOTCH main study protocol originally had a target of 468 participants (234 of whom will be allocated to the intervention) and anticipated running 24 groups. To ensure a random sample of groups for the fidelity study a statistician using a random number generator will identify; three Day 1 sessions, three Day 2 sessions and three Day 3 sessions from early, middle and late stages of the study. This will ensure we listen to approximately 10% of group sessions across the duration of the study. It was not possible to listen to all the sessions due to pragmatic reasons of time and cost. Through extensive discussions with the team who developed the intervention we decided to pre-specify those sessions which were considered by the team to be key to promoting behaviour change and contain either educational or discussion items. Other sessions which are more practical in nature (e.g. origami for distraction or relaxation) will be difficult to assess from an audio recording as the aim is to promote distraction and discussion or experience a relaxation technique. These will be checked to see if they took place as a minimum requirement of the intervention but will not be rated for facilitator adherence and competence. See table 4.

For peer review only - http://bmjopen.bmj.com/site/about/guidelines.xhtml

For peer review only

Table 4 Course programme with sessions identified for fidelity

**DAY 1**
Session 1 Introduction
***Session 2 Pain information**
***Session 3 Painkiller information and opioid education**
***Session 4 Acceptance: John's story**
Session 5 Attention Control and distraction
Session 6 Distraction activity – rose drawing
***Session 7 Good days, Bad days when is pain bearable and when is it not?**
***Session 8 The pain cycle unhelpful emotions and behaviours**
Session 9 Posture
Session 10 Relaxation and Breathing
Session 11 Summary of the day
**DAY 2**
Session 12 Reflections from day 1
***Session 13 Stress-busting – prioritising what's important, action planning, goal setting and pacing**
***Session 14 Withdrawal symptoms, case studies (Opioid Education 2)**
Session 15 Distraction activity- origami
***Session 16 Identifying and overcoming Barriers to change Part 1 – recognising unhelpful thinking**
***Identifying and overcoming Barriers to change Part 2– reframing negatives to positives**
Session 17 Mindful attention control
Session 18 Balance and introduction to stretch
Session 19 Summary of the Day
**DAY 3**
Session 20 Reflections from day 2 and previous week
***Session 21 Anger, irritability and frustration**
***Session 22 Relationships Part 1 Getting the most from your healthcare team**
Session 22 Part 2 Relationships Part 2 Listening skills
***Session 23 Managing setbacks and non-drug management techniques**
Session 24 Distraction activity – mindfulness colouring
Session 25 Stretching muscles that commonly get tight
Session 26 Mindfulness of thoughts and Senses
Session 27 Summary of Day 3
Session 28 Summary of the course

Legend	***Educational and/or self-management regarding pain or opioid use**	Practical, reflection or summarising sessions
Day 1	**2, 3, 4, 7, 8**	1,5,6,9,10,11
Day 2	**13,14,16**	12,15,17,18,19
Day 3	**21,22 part 1,23,**	20,22 part 2, 24,25,26,27,28

To assess the fidelity of the intervention we will assess two aspects of intervention delivery; adherence to the intervention manual and competency of the facilitators. A member of the process evaluation team (VN) will listen to the relevant recordings and score adherence and competence using a specially devised checklist based upon components specified in the manual items and training protocol for intervention facilitators. See appendices 2 and 3 for examples.

We will also rate one of the first or second one-to-one nurse consultations per group N=24. We will double rate 10% of these sampled group and one-to-one sessions by a second member of the team (KS) to assess

For peer review only - http://bmjopen.bmj.com/site/about/guidelines.xhtml

inter-rater reliability and to ensure rigour. Percentage scores will be given for adherence and competence per session and the findings analysed using standard statistical methods. (See mixed methods analysis)

3) Change Mechanisms assessing whether hypothesised change occurred.

We will administer self-report questions within the I-WOTCH RCT questionnaires (at baseline, four, eight, and twelve months) to track possible change mechanisms. Specifically we will assess participants' (i) motivation to reduce opioid use before and after the intervention, (ii) expectations of success in opioid reduction and (iii) confidence (or self-efficacy (10)) in relation to opioid reduction prior to receiving the intervention and (iv) perceived intervention efficacy before and after participation, see table 5. These data will be analysed using standard statistical methods, including t tests and ANCOVA. The technical issues of the statistical analyses will be detailed in the overall trial statistical analysis plan.

Table 5 Motivation Expectations, Self-Efficacy and Perceived Intervention Efficacy Questions

**Baseline Motivation** (baseline and follow up)<br>**I want to reduce my opioid use**<br>(Not at all, by a little, by half, so I only use a little, so I use no opioids)<br><br>**Baseline Expectation** (baseline only)<br>**I expect that, in 4 months' time, I will have reduced my opioid use**<br>(Not at all, by a little, by half, so I only use a little, so I use no opioids)<br><br>**Baseline Self-Efficacy** (baseline only)<br>**I am confident I could reduce my opioid use a lot over 4 months**<br>(Not at all confident, somewhat confident, fairly confident, strongly confident, completely confident)<br><br>**Perceived Intervention Efficacy** (baseline and follow up)<br>**Baseline**<br>**I feel that involvement in this study can help me to reduce my opioid use**<br>Not at all, by a little, by half, so I only use a little, so I use no opioids<br><br>**Follow up**<br>**I feel that involvement in this study has helped me to reduce my opioid use**<br>Not at all, by a little, by half, so I only use a little, so I use no opioids

For peer review only - http://bmjopen.bmj.com/site/about/guidelines.xhtml

4) Contextual issues that may affect the outcome or running of the study and/or intervention

Contextual factors may be found in the data collected above may influence change and outcomes. We will explore this as the need arises from the data as it may be a ‘thread to follow’ (see paragraph below) or an integral part of a section of the analysis.

Mixed Methods Analysis

Quantitative data will be analysed statistically to produce appropriate descriptive statistics, tables, charts or figures. Data from quantitative and qualitative findings will be integrated as outlined by O’Cathain et al.(11) We will use both ‘following a thread’ which involves selecting a question or component from one aspect of the findings and following across, and ‘mixed methods matrix’ where, for example, responses on quantitative scales can be compared to interview transcript, and data on each case can be concisely stated and recorded on a matrix. For detailed explanation of ‘following a thread’, we refer the reader to O’Cathain.(11)

Patient and Public Involvement

This process evaluation is part of the I-WOTCH study which has patient and public involvement with regard to input into its design as well as the ongoing running of the study which is described more fully elsewhere.(1) Patient participant interviews are an integral part of this process evaluation. All trial participants will be notified of the study findings via a study newsletter and a lay summary will be available on the study website.

Trial Status

The I-WOTCH study began recruitment in May 2017 and anticipate groups will be running into February 2019, data collection will be completed around February 2020 and they expect the final report for the funders will be submitted mid-2020.

For peer review only - http://bmjopen.bmj.com/site/about/guidelines.xhtml

Ethics and Dissemination

We intend to publish the process evaluation findings in peer reviewed journals and details of the main trial ethics and dissemination are outlined in the main trial protocol.(1)

The I-WOTCH trial and process evaluation have been granted full ethics approval by Yorkshire & The Humber - South Yorkshire Research Ethics Committee on September 13th, 2016 (16/YH/0325).

References

1. Sandhu HK, Abraham C, Alleyne S, et al. Testing a support programme for opioid reduction for people with chronic non-malignant pain: the I-WOTCH randomised controlled trial protocol. BMJ Open2019;9(8):e028937.
2. Moore GF, Audrey S, Barker M, et al. Process evaluation of complex interventions: Medical Research Council guidance. 2015;350.
3. Steckler A, Linnan L. Process Evaluation for Public health Interventions and research. San Fransisco: Jossey-Bass; 2002.
4. Basch CE, Sliepcevich EM, Gold RS, et al. Avoiding type III errors in health education program evaluations: a case study. Health Educ Q1985;12(4):315-31.
5. Bartholomew Eldredge LK, Markham CM, Ruiter RAC, et al. Planning health promotion programs: Jossey-Bass Inc.; 2016 2016.
6. Borek AJ, Abraham C, Smith JR, et al. A checklist to improve reporting of group-based behaviour-change interventions. BMC Public Health2015;15:963.
7. Fisher JDF, W.A. The Information-Motivation-Behavioural Skills Model. In: DiClemente R, Crosby R, Kegler M, editors. Emerging theories in health promotion practice and research: Strategies for improving public health. San Fransisco: Jossey-Bass; 2002. p. 40-7.
8. Ritchie J, Spencer L. Qualitative data analysis for applied policy research. The qualitative researcher's companion2002;573(2002):305-29.
9. Braun V, Clarke V. Using thematic analysis in psychology. Qualitative Research in Psychology2006;3(2):77-101.
10. Bandura A. Self-efficacy: toward a unifying theory of behavioral change. Psychol Rev1977;84(2):191-215.
11. O'Cathain A, Murphy E, Nicholl J. Three techniques for integrating data in mixed methods studies. BMJ (Online)2010;341(7783):1147-50.

Authors' contributions

KS, MU, SE and HS conceived the original study design. VN, KS and CA have developed the study design, and plan for data collection and analysis. All authors have provided critical revisions to the manuscript and approved the final manuscript.

For peer review only - http://bmjopen.bmj.com/site/about/guidelines.xhtml

Funding

This project is funded by the National Institute for Health Research (NIHR), Health Technology Assessment (HTA) (project number 14/224/04). The views and opinions expressed therein are those of the authors and do not necessarily reflect those of the HTA, NIHR, NHS or the Department of Health.

Competing interests statement.

SE is investigator on a number of NIHR and industry sponsored studies. He received travel expenses for speaking at conferences from the professional organisations. SE consults for Medtronic, Abbott, Boston Scientific and Mainstay Medical, none in relation to opioids. SE is chair of the BPS Science and Research Committee. SE is deputy Chair of the NIHR CRN Anaesthesia Pain and Perioperative Medicine National Specialty Group. SE's department has received fellowship funding from Medtronic as well as nurse funding from Abbott.

HS is director of Health Psychology Services Ltd, providing psychological services for a range of health related conditions.

MU was Chair of the NICE accreditation advisory committee until March 2017 for which he received a fee. He is chief investigator or co-investigator on multiple previous and current research grants from the UK National Institute for Health Research, Arthritis Research UK and is a co-investigator on grants funded by the Australian NHMRC. He is an NIHR Senior Investigator. He has received travel expenses for speaking at conferences from the professional organisations hosting the conferences. He is a director and shareholder of Clinvivo Ltd that provides electronic data collection for health services research. He is part of an academic partnership with Serco Ltd related to return to work initiatives. He is a co-investigator on a study receiving support in kind from Orthospace Ltd. He is an editor of the NIHR journal series, and a member of the NIHR Journal Editors Group, for which he receives a fee.

KS received grant funding as PI and CoI from NIHR for other projects and was on the NIHR HS&DR Funding Board until January 2018.

For peer review only

Acknowledgements

This manuscript was written on behalf of the I-WOTCH team:

Sharisse Alleyne, University of Warwick
Shyam Balasubramanian, Department of Anaesthesia & Pain Medicine University Hospitals Coventry and Warwickshire NHS Trust
Lauren Betteley, University of Warwick
Katie Booth, University of Warwick
Dawn Carnes, Barts & The London Queen Mary's School of Medicine & Dentistry
Andrea Dompieri Furlan, Toronto Rehabilitation Institute, University Health Network, Canada
Kirstie Haywood, University of Warwick
Ranjit Lall, University of Warwick
Andrea Manca, The University of York
Dipesh Mistry, University of Warwick
Jennifer Noyes, The James Cook University Hospital
Anisur Rahman, University College London
Jane Shaw, The James Cook University Hospital
Nicole Kit Yee Tang, University of Warwick
Stephanie Taylor, Barts & The London Queen Mary's School of Medicine & Dentistry
Colin Tysall, University/User Teaching and Research Action Partnership, University of Warwick
Emma Withers, University of Warwick
Martin Underwood, University of Warwick
Cynthia Paola Iglesis Urrutia, The University of York

Figure 1 Logic Model

Figure 2 Information Motivation Behavioural skills model

For peer review only

For peer review only - http://bmjopen.bmj.com/site/about/guidelines.xhtml

Figure 1 Logic model

The problem	Intervention Aims	Intervention	Theory and Guidance	Interim Targets	Desired Outcomes
People with chronic non-malignant pain are taking opioids, which have side effects and are not effective in the long term.	To test the effectiveness and cost effectiveness of a patient-centred multicomponent self-management intervention targeting withdrawal of strong opioids on activities of daily living for people living with chronic non-malignant pain	**Manualised Intervention Delivery**<br>**Core pain management topics:**<br>• Acute versus Chronic pain<br>• Acceptance<br>• Attention Control and distraction<br>• the pain cycle<br>• Posture and movement advice<br>• Relaxation techniques<br>• Stress busting for health action planning, problem solving, pacing, SMART goal setting<br>• identifying and overcoming barriers to change<br>• Mindfulness<br>• Anger, irritability and frustration<br>• Communication Skills<br><br>**Core opioid specific topics:**<br>• The rationale of prescribing in chronic pain<br>• Opioid induced tolerance and need for dose escalation<br>• Evidence of usefulness of opioids short and long term<br>• Side effects of opioids short term and long term<br>• Case studies of successful discontinued opioid therapy<br>• Opioid withdrawal symptoms<br>• Advantages of slow supervised tapering<br>• Symptom management during tapering<br>• Pain control after opioids	Theory of Planned Behaviour<br><br>Social Cognitive Theory<br><br>Information Motivation and Behavioural (IMB model) skills<br><br>Patient Centred Communication<br><br>Motivational Interviewing	**Staff Training**<br>To facilitate groups, deliver individual tapering consultations and telephone support in an inclusive and non-judgemental manner<br>**Individual participant changes:**<br>a Knowledge of: opioids, withdrawal effects, chronic pain<br>b Fostering change: self-validation, legitimising pain, normalising expectations<br>c Motivation to change by: Improved self-efficacy, effective tapering<br>d Skills:<br>• General Self-Regulation<br>*Psychological skills*<br>*Identify reasons for negative emotions (anger /frustration /irritable)*<br>*Identify problems and solutions, barriers to change*<br>*Recognise errors in thinking/automatic thoughts*<br>*Goal setting, goal review*<br>*Physical skills*<br>*Promote body awareness, posture*<br>*Reduce muscle tension*<br>*Body awareness and core strength*<br>*Relaxation-contract relax*<br>• Pain Self-Regulation<br>Understand that pain and mood are linked – when is pain bearable and when not bearable.<br>Understanding of pain cycle, unhelpful emotions and behaviours<br>Using mind to relieve pain does not mean pain in mind<br>Distraction whilst relaxed<br>Focus mind away from pain<br>Mindfulness for pain<br>Managing flare ups<br>Need for stretching<br>• Communication Skills<br>How to communicate with General Practitioners (GPs) and Health Care Professionals (HCPs)<br>Listening skills - Active and giving feedback in communication-reward for help.	**Primary outcomes:**<br>Patient-Reported Outcomes Measurement Information System (PROMIS) Pain Interference Short Form (8A)(PROMIS-PI-SF-8A)<br><br>Daily morphine equivalent opioid dose

For peer review only

Figure 2. The Information-Motivation-Behavioural Skills Model

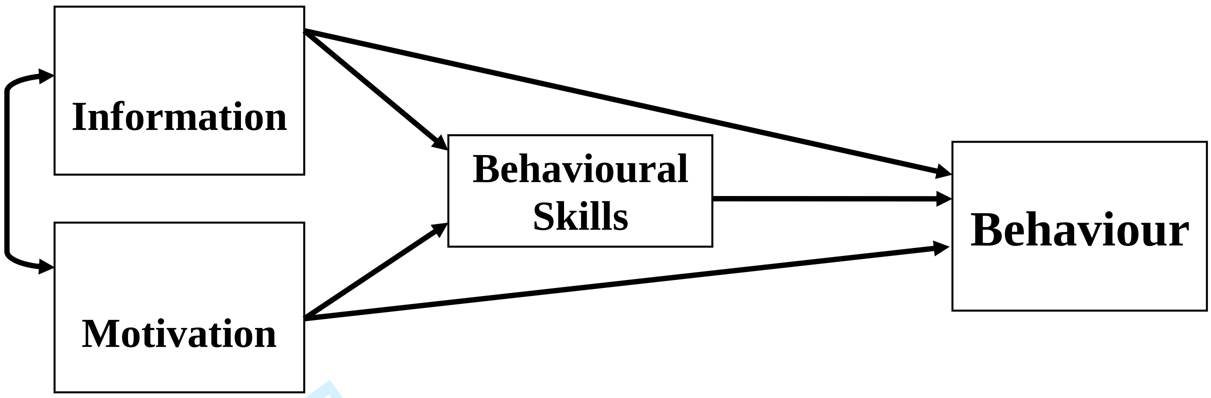

From J. D. Fisher and W. A. Fisher (1992). Changing AIDS risk behavior. Psychological Bulletin, 111, 455–74. Copyright by APA. Reprinted with permission.

For peer review only

Group venue: ______________________________

Date: ______________________________

I-WOTCH Feedback form

Thank you for attending the I-WOTCH course. Please complete the feedback and short questionnaire below. This information will be used to evaluate the support programme. Please note that these forms are anonymous and will be sent back to the study team.

1. Were the aims of the course made clear? Please circle one

Yes *No* *Don't know*

2. What were the three most useful things on this course?

1)..

2)..

3)..

3. What three things would you suggest to make this course better for future participants?

1) ....

2) ....

3) ....

4. How confident do you feel that the course content will help you personally? *(Please circle one)*

Very confident *Confident* *Not very confident* *Not confident at all*

5. How confident do you feel that you will be able to use this in the future? *(Please circle one)*

Very confident *Confident* *Not very confident* *Not confident at all*

PLEASE TURN OVER THE PAGE FOR REMAINING QUESTIONS

IWOTCH Participant Feedback Form V1.0 09Oct18. IRAS reference: 199154

This project was funded by the National Institute for Health Research, Health Technology Assessment (project number 14/224/04).
The views and opinions expressed therein are those of the authors and do not necessarily reflect those of the HTA, NIHR, NHS or the Department of Health.

Funded by NIHR

Group venue: ______________________________

Date: ______________________________

6. Overall were the facilitators: *(Please circle one)*

Very good *Good* *Satisfactory* *Poor*

7. Overall were the handouts: *(Please circle one)*

Very good *Good* *Satisfactory* *Poor*

8. How did you find the face to face meeting with the nurse? *(Please circle one)*

Very useful *Useful* *Not very useful* *Not useful at all*

9. How did you find the telephone calls with the nurse? *(Please circle one)*

Very useful *Useful* *Not very useful* *Not useful at all*

10. Overall how useful did you find the whole course? *(Please circle one)*

Very useful *Useful* *Not very useful* *Not useful at all*

11. Is there anything else you would like to say?

Thank you.

Please return in the stamped addressed envelope

IWOTCH Participant Feedback Form V1.0 09Oct18. IRAS reference: 199154

This project was funded by the National Institute for Health Research, Health Technology Assessment (project number 14/224/04).
The views and opinions expressed therein are those of the authors and do not necessarily reflect those of the HTA, NIHR, NHS or the Department of Health.

For peer review only - http://bmjopen.bmj.com/site/about/guidelines.xhtml

DAY 1 I-WOTCH Fidelity checklists

Group ID: **Date of group session:**

**Day 1: Living with and dealing with pain**	**Date listened**	**Occurred/ Did not**	**Adherence score**	**Competence score**	**Comments**
Session 1 Introduction 15mins					
***Session 2<br>Pain information approx. 45mins**					
***Session 3<br>Painkiller information and opioid education approx. 45mins**					
Break					
***Session 4<br>Acceptance: John's story 20mins**					
Session 5 Attention control and distraction 10mins					
Session 6 Distraction activity – rose drawing 20mins					
Lunch					
***Session 7<br>Good days, Bad days when is pain bearable and when is it not? 40mins**					
***Session 8<br>The pain cycle unhelpful emotions and behaviours 40mins**					
Break					
Session 9 Posture					
Session 10 Relaxation and Breathing					
Session 11 Summary of the day					
**Total scores**					

Comments: *e.g. session not recorded so subsequent group same session listened to instead*

For peer review only

I-WOTCH course code: _____ Reviewer: _________________________ Review date: ____________

Day 1 /Session 2 /Title: Pain Information 30mins

Adherence: of the delivery as per protocol

No.	Item	Adherence	Comments
Intro	Did the facilitator(s) introduce the session?	Yes (2) Partially (1) No (0)	
Step 1	Did the facilitator(s) play the DVD of the biomedical explanation about acute and chronic pain?	Yes (2) Partially (1) No (0)	
	Did the facilitator(s) ask the group Q1 and discuss, "What do you think about this explanation of pain? Is it missing anything?"	Yes (2) Partially (1) No (0)	
Step 2	Did the facilitator(s) present the bio-psycho-social explanation of pain?	Yes (2) Partially (1) No (0)	
	Did the facilitator(s) ask the group Q2 and discuss, "What do you think about this explanation of pain?"	Yes (2) Partially (1) No (0)	
Step 3	Did the facilitator(s) play the DVD of Experiences of living with opioid- treated long term pain?	Yes (2) Partially (1) No (0)	
	Did the facilitator(s) ask the group Q3 and discuss, "What do you think about Caroline's description of living with opioid-treated long-term pain?"	Yes (2) Partially (1) No (0)	
Summary	Did the facilitator(s) consolidate/embed the group's learning at the end of the session? *e.g. reading the summary, putting the session in context*	Yes (2) Partially (1) No (0)	
	Total adherence score (max 16)		
	Percentage adherence score (Total adherence score */16x100)		

Instructions:

When at all possible please rate as 'Yes' or 'No' If 'partially' then write reason in comments box
Questions need not be verbatim (unless specified) as long as content of session is covered.

For peer review only

I-WOTCH course code: _____ **Reviewer:** __________________________ **Review date:** ____________

Comments: For use if sessions; go off track, include items which are not on checklist, contain surprising unforeseen aspects or the facilitation wasn't covered as intended. Also if there was no opportunity to demonstrate the skill listed.

For peer review only

I-WOTCH course code: _____ Reviewer: __________________________ Review date: ____________

Day 1 /Session 2 /Title: Pain Information and Opioid Education

Competence of the quality of delivery or 'skill' of the facilitators

	Item	Competence measure	Comments (*use box below to expand*)
1	Did the facilitator(s) create opportunities for discussion *e.g. did they; encourage individuals to participate, ask open questions, give enough time for the group to answer (rather than answer their own questions)*	Evident (2)<br>Partially evident (1)<br>Not evident (0)<br>Did not happen in this session (N/A)	
2	Did the facilitator encourage active participation across group members? *e.g. did they encourage quieter members and manage dominant members?*	Evident (2)<br>Partially evident (1)<br>Not evident (0)<br>Did not happen in this session (N/A)	
3	Did the facilitator(s) encourage individual disclosure? *e.g. did they ask different group members to comment or encourage the group to explore issues further (either individually or as a group)?*	Evident (2)<br>Partially evident (1)<br>Not evident (0)<br>Did not happen in this session (N/A)	
4	Did the facilitator(s) validate participants' disclosures? *e.g. Do other people find this/think that? I know how you feel. Sometimes people may feel differently about things.*	Evident (2)<br>Partially evident (1)<br>Not evident (0)<br>Did not happen in this session (N/A)	
5	Did the facilitator(s) give encouraging feedback on participants reported behaviours? *e.g. Did they give appraisal 'that's really good' or 'that's really good but I wonder if…'*	Evident (2)<br>Partially evident (1)<br>Not evident (0)<br>Did not happen in this session (N/A)	
6	Did the facilitator(s) foster a positive group climate? *e.g. did they; use humour, say positive things about people 'that's a helpful comment' 'thank you for sharing that'*	Evident (2)<br>Partially evident (1)<br>Not evident (0)<br>Did not happen in this session (N/A)	
7	Did the facilitator acknowledge and respond appropriately to admissions or statements of low self-efficacy? *e.g. 'yes this can be difficult but…' ideas or examples offered of how this may be done.*	Evident (2)<br>Partially evident (1)<br>Not evident (0)<br>Did not happen in this session (N/A)	
8	Did the facilitator respond appropriately to disclosures of negative events?	Evident (2)<br>Partially evident (1)<br>Not evident (0)<br>Did not happen in this session (N/A)	

For peer review only

I-WOTCH course code: _____ **Reviewer: _________________________** **Review date: ____________**

	Total competence score (max 16)		
	Percentage competence score		

Comments: For use if sessions; go off track, include items which are not on checklist, contain surprising unforeseen aspects or the facilitation wasn't covered as intended. Also if there was no opportunity to demonstrate the skill listed.

For peer review only
